# Avian coronaviruses induce inflammatory responses by activating p38/MAPK signaling and NLRP3/caspase-1 inflammasomes through sphingosine-1-phosphate receptor 1

**DOI:** 10.1186/s13567-026-01768-0

**Published:** 2026-05-23

**Authors:** Hongtao Xiao, Siyao Yu, Shijing Li, Zhifei Xu, Jiayang Chen, Lijin Lai, Qiuyan Lin, Tao Ren, Libin Chen

**Affiliations:** 1 College of Veterinary Medicine, South China Agricultural University, Guangzhou, People’s Republic of China; 2 Key Laboratory of Animal Vaccine Development, Ministry of Agriculture and Rural Affairs, Guang zhou, People’s Republic of China; 3 National and Regional Joint Engineering Laboratory for Medicament of Zoonosis Prevention and Control, Guangzhou, People’s Republic of China; 4 Key Laboratory of Zoonosis Prevention and Control of Guangdong Province, Guangzhou, People’s Republic of China

**Keywords:** Metabolomic analysis, inflammatory response, sphingosine-1-phosphate receptor 1, infectious bronchitis virus, cytokine regulation, mitogen-activated protein kinase signaling

## Abstract

Infectious bronchitis virus (IBV), a member of the γ-coronavirus genus within the Coronaviridae family, is a major pathogenic threat to the global poultry industry. Most IBV infections induce severe renal pathological lesions in chickens, yet the renal metabolic perturbations triggered by IBV infection remain largely elusive. Herein, we applied a metabolomic approach to characterize the metabolic profiles of kidney tissues from IBV-infected specific-pathogen-free (SPF) chickens. IBV infection caused profound alterations in the renal metabolome, encompassing amino acids and their derivatives, energy metabolites, and lipid molecules. Most amino acids and their derivatives were significantly downregulated at 7 days post-infection (dpi), indicating that IBV usurps host amino acid pools to support its own replication in SPF chickens. In addition, the levels of L-glutamine, D-mannose 1-phosphate, and D-galacturonate were markedly elevated post-infection, implying their potential roles in mediating host energy utilization and facilitating viral replication during IBV infection. Subsequently, sphingosine-1-phosphate (S1P)—a key bioactive lipid molecule—was identified as a prominently upregulated metabolite in IBV-infected kidney tissues. Follow-up functional experiments revealed that IBV infection upregulates the S1P-S1PR1 signaling axis, and concomitantly activates the p38/JNK/MAPK pathway as well as the NLRP3/caspase-1 inflammasome. Notably, S1PR1 was found to modulate the p38/MAPK signaling pathway and NLRP3/caspase-1 inflammasome, thereby regulating the expression of the proinflammatory cytokines IL-1β and IL-18. Collectively, our study delineates the comprehensive renal metabolic landscape of chickens in response to IBV infection and identifies S1PR1 as a pivotal regulatory target of IBV-induced renal inflammation. These findings provide novel mechanistic insights for the development of preventive and therapeutic strategies targeting metabolic signaling pathways against IBV infection.

**Supplementary Information:**

The online version contains supplementary material available at 10.1186/s13567-026-01768-0.

## Introduction

Infectious bronchitis virus (IBV) belongs to the γ-coronavirus family and is an enveloped, single-stranded positive-sense RNA virus with a genome size of approximately 27.6 kb. IBV was first identified in the USA in the early 1930s, with the respiratory tract of both young and adult chickens serving as the primary target organ [1]. Similar to other coronaviruses, IBV undergoes continuous genomic changes through mutations and recombination, resulting in significant variation in clinical symptoms and pathological manifestations among different strains in infected animals. Over the years, multiple strains associated with reproductive, renal, gastrointestinal, muscular, and immunosuppressive diseases have emerged, causing substantial economic losses in global poultry production. Renal-tropic IBV strains can infect renal ciliated cells, including those in the proximal, distal, and collecting ducts, leading to weight loss, watery feces, increased water consumption, and elevated mortality. These strains can also spread from the respiratory tract via blood monocytes [2, 3]. Infectious bronchitis (IB) has now become a global disease, with multiple genotypes or serotypes of IBV coexisting for long periods within the same region [4–7].

Viral infection relies on host cell biosynthesis to complete critical steps in the infection cycle, including invasion, replication, viral particle assembly, and the release of progeny viruses. During this process, viruses hijack key biomolecules from host metabolic pathways, such as amino acids, carbohydrates, and lipids, to obtain the raw materials and energy needed for replication [8]. Thus, viral infection and replication are closely linked to the metabolic behavior of host cells. Metabolomics, a novel approach for identifying disease-specific small metabolites in tumors and various other conditions, has been extensively applied in virology research [9, 10]. African swine fever virus (ASFV) reprograms host cell metabolism and regulates host nucleotide biosynthetic pathways to generate the essential nucleotide substrates required for its replication [11]. Porcine reproductive and respiratory syndrome virus utilizes the ROS/HIF-1α axis to reprogram host glucose metabolism via the pentose phosphate pathway, creating a biosynthetic environment conducive to viral propagation [12]. Following infection with the highly pathogenic avian influenza H5N1 virus, key metabolites such as sphingosine, psychosine sulfate, and L-serine—each involved in viral endocytosis and cellular signaling—undergo significant alterations in infected chickens [13]. These studies indicate that, on the one hand, viral infections hijack amino acids, nucleotides, and energy molecules produced in host metabolic pathways for their own replication. On the other hand, the host immune system modulates metabolic responses to counteract metabolic damage caused by viral infection. In summary, alterations in metabolic responses reflect the progression of viral infection and the stages of host injury. Understanding how viral infection affects host cell metabolism will enhance our knowledge of pathogenic mechanisms and facilitate the development of control measures targeting critical metabolic pathways.

During virus–host interactions, pattern recognition receptors and other proteins act as key sensors of viral infection, coordinating immune responses while reprogramming host metabolism to sustain antiviral defenses [14]. However, several viruses exploit these metabolic changes to enhance replication, evade immune surveillance, and modulate cytokine production. Metabolomic analysis of the bursa of Fabricius in chickens infected with nephropathogenic IBV revealed 75 significantly differentially expressed metabolites, and these metabolic biomarkers showed strong correlations with inflammatory cytokines [15]. The effect of IBV infection on renal metabolites, the primary target organ following IBV infection, remains unknown. To facilitate replication within host cells, viruses can hijack organelles to synthesize metabolites required for their replication. Therefore, elucidating the relationship between these significantly altered metabolites and the pathological effects induced by viral infections is crucial for understanding viral infection mechanisms.

In a previous study, we isolated a kidney-tropic GI-7 lineage of IBV and characterized its genetic diversity and evolutionary dynamics during transmission [16]. Although the GI-19 lineage of IBV represents a major proportion of isolates in China, the isolation rate of the GI-7 lineage has steadily increased in recent years, posing a serious threat to poultry health [17–19]. In this study, we collected kidneys from specific pathogen-free (SPF) chickens infected with the GI-7 strain of IBV at 7 days post-infection and employed nontargeted metabolomics to detect and analyze differential metabolites in the kidneys. The results revealed significant metabolic alterations in IBV-infected kidneys, with amino acid metabolomics showing a decline in most amino acids and their derivatives due to viral utilization. Furthermore, Kyoto Encyclopedia of Genes and Genomes (KEGG) enrichment analysis revealed significant alterations in S1P within immune-related lipid metabolic pathways. Subsequent experiments confirmed that the S1P target receptor S1PR1 plays a crucial role in IBV-mediated inflammatory processes. Our findings elucidate the changes in renal metabolic profiles following IBV infection and identify S1PR1 as a potential therapeutic target for the clinical prevention and control of IBV.

## Materials and methods

### Viruses and cells

IBV 220198GDZC (GI-7) and 250607GXCX (GVII-1) were isolated and stored by this laboratory. The Beaudette strain is maintained in this laboratory. The Beaudette strain is a cell-adapted strain derived from multiple passages through chicken embryos and cells [53]. 250607GXCX (GVII-1) is an IBV strain verified by our research group to possess cytopathic infectivity [54]. 220198GDZC (GI-7) has been confirmed to infect DF-1 cells via indirect immunofluorescence assay (data not shown). DF-1 cells (immortalized chicken embryo fibroblasts, UMSNSAH/DF-1) were stored in the laboratory and cultured in DMEM (Gibco) supplemented with 10% FBS in a 5% CO_2_ incubator.

### SPF chickens

SPF white leghorn chickens and SPF embryonated eggs were purchased from Guangdong Dahuanong Poultry & Egg Co. (Guangzhou, China). Our study was approved by the Animal Welfare and Ethical Censor Committee of South China Agricultural University.

### Chemicals and antibodies

The S1PR1-specific inhibitor W146 (sc 296700) was purchased from Santa Cruz Biotechnology (USA). The drug solvent dimethyl sulfoxide (DMSO) and the S1PR1-specific agonist SEW2871 (S3944) were purchased from Sigma (USA). Rabbit anti-chicken NLRP3 polyclonal antibody and rabbit anti-S1PR1 (EDG-1) antibody (BS2593) were purchased from BioWorld (USA). Rabbit anti-GAPDH antibody (ab181602) was purchased from Abcam (UK). Rabbit anti-phospho-p38/MAPK antibody (#4511) and rabbit anti-p38/MAPK antibody (#8690) were purchased from Cell Signaling Technology (USA). Rabbit anti-phospho-JNK1/MAPK antibody and rabbit anti-JNK1/MAPK antibody were purchased from Proteintech (China). IBV N protein monoclonal antibody was purchased from GeneTex (USA).

### Sample collection

Ten SPF chickens were infected with IBV 220198GDZC (GI-7), while the control group received Phosphate buffered saline (PBS) inoculation; 7 days post-infection, kidneys and serum samples were aseptically collected from each chicken. Kidneys were placed directly into cryovials and rapidly frozen in liquid nitrogen for metabolite extraction. Serum samples were centrifuged at 3000 rpm for 10 min to remove red blood cells. The supernatant was aspirated and stored at −80 °C.

### Metabolite extraction

Metabolite extraction happens as follows: take 0.1 mg of the kidney sample to be tested, grind it in liquid nitrogen, add 100 µL of ultrapure water, and place it in an EP tube. Add 400 µL of 80% methanol aqueous solution, vortex mix, and incubate at −20 °C for 60 min. Centrifuge at 14 000 *g*, 4 °C for 20 min. Transfer a measured volume of supernatant to a 1.5 mL centrifuge tube. Freeze-dry under vacuum. Resuspend the residue in 100 µL of the reconstitution solvent, vortex, and centrifuge at 14 000 *g*, 4 °C for 15 min. Collect the supernatant for liquid chromatography-mass spectrometry (LC–MS) analysis. Take an equal volume of supernatant from each processed sample, mix thoroughly, and use as a quality control (QC) sample. The blank sample is the blank matrix of the experimental sample, with the same sample preparation process as the experimental samples.

### Data acquisition through LC–MS analysis

Data acquisition was performed using LC–MS analysis on pretreated experimental samples. Chromatographic conditions were as follows: column: Accucore HILIC column; column temperature: 40 °C; flow rate: 0.3 mL/min; normal-mode mobile phase A: 0.1% formic acid, 95% acetonitrile, 10 mM ammonium acetate; normal mode mobile phase B: 0.1% formic acid, 50% acetonitrile, 10 mM ammonium acetate; negative mode mobile phase A: 95% acetonitrile, 10 mM ammonium acetate, pH 9.0; negative mode mobile phase B: 50% acetonitrile, 10 mM ammonium acetate, pH 9.0. Mass spectrometry analysis conditions are as follows: scan range selected at m/z 100–1500; ESI source settings: Spray Voltage 3.2 kV, sheath gas flow rate 35 arb, aux gas flow rate 10 arb, capillary temp 320 °C; MS/MS secondary scanning performed as data-dependent scans.

Import the raw data (.raw) files into the CD search library software for preliminary screening based on parameters such as retention time and mass-to-charge ratio. Then align peaks across different samples using a retention time deviation of 0.2 min and a mass deviation of 5 ppm to enhance identification accuracy. Subsequently, perform peak extraction using configured parameters including a mass deviation of 5 ppm, signal intensity deviation of 30%, signal-to-noise ratio of 3, minimum signal intensity of 100 000, and sum ions. Peak areas were quantified, target ions were integrated, and molecular formulas were predicted using molecular and fragment ions. These were compared against mzCloud, mzVaulth, and MassList databases; background ions were removed; quantitative results were normalized; and final identification and quantification results were obtained.

### Amino acid metabolism analysis

The liquid chromatography conditions primarily include: column: ACQUITY BEH Amide column (1.7 µm, 100 mm × 2.1 mm i.d.); mobile phase: phase A: ultra-pure water (containing 2 mM ammonium acetate, 0.04% formic acid); mobile phase: phase A, ultrapure water (containing 2 mM ammonium acetate, 0.04% formic acid); phase B, acetonitrile (containing 2 mM ammonium acetate, 0.04% formic acid); gradient elution program: 0–1.2 min A/B = 10:90 (V/V), 9 min A/B = 40:60 (V/V), 10–11 min: 60:40 (v/v), 11.01–15 min: 10:90 (v/v); flow rate: 0.4 mL/min; column temperature: 40 °C; injection volume: 2 μL.

Mass spectrometry conditions primarily include: electrospray ionization (ESI) source temperature at 550 °C, mass spectrometer voltage of 5500 V in positive ion mode, mass spectrometer voltage of −4500 V in negative ion mode, and curtain gas (CUR) at 35 psi. In the Q-Trap 6500 + , each ion pair is scanned and detected on the basis of optimized declustering potential (DP) and collision energy (CE).

### Gene knockdown experiment

DF-1 cells were seeded at 40% confluence in 6-well plates. In addition, 5 μL of siRNA was mixed with 250 μL of cell culture medium and 2.5 μL of lipo2000 transfection reagent (Thermo Fisher) was mixed with 250 μL of cell culture medium and incubated for 5 min. The two reagent mixtures were combined, mixed thoroughly, and incubated at room temperature for 20 min before being added dropwise to the cells.

### ELISA

According to the manufacturer’s instructions, the expression of inflammatory cytokines in cell supernatants and kidneys was detected using an enzyme-linked immunosorbent assay (ELISA) kit for chicken interleukin (IL)-18 and IL-1β (Jiangsu Meibiao Biotechnology Co., Ltd.). Chicken L-Glutamine, D-Mannose 1-phosphate, and D-galacturonate were detected in chicken serum and kidney samples using ELISA kits (Jiangsu Meibiao Biotechnology Co., Ltd.). Sample measurements were calculated on the basis of the measurements of the standard substances.

### Viral infection, drug treatment, and cell viability assay

DF-1 cells were seeded in 35 mm cell culture dishes. When cell confluence reached 90%, the culture medium was discarded and cells were washed twice with PBS. DF-1 cells were then pretreated with 1 mL of S1PR1-specific agonist SEW2871 (0.5 μM/mL) and S1PR1-specific antagonist W146 (10 μM/mL) dissolved in dimethyl sulfoxide (DMSO) [49]. Cells were inoculated with an equal volume of DMSO as a control. After incubation for 1 h, the original solution was removed, and cells were infected with IBV at a multiplicity of infection (MOI) of 1. Following infection, cell culture supernatants and cells were collected as required for subsequent experiments. Cell viability was assessed using the CCK-8 Cell Proliferation and Cytotoxicity Detection Kit (AbMole Bioscience, Houston, TX, USA) according to the manufacturer’s instructions. Cells were seeded at a density of 5 × 10^3^ DF-1 cells per well in 96-well plates. Following treatment with W146, SEW2871, or dimethyl sulfoxide (DMSO), 10 μl of CCK-8 reagent was added and incubated at 37 °C for 2 h. Absorbance was measured at 450 nm as the reference wavelength.

### Fluorescent quantitative PCR

Total RNA was extracted from cells using Freezol reagent (Vazyme). The extracted RNA was used to detect the copy number of the target gene via one-step absolute quantitative polymerase chain reaction (qPCR) (Vazyme). Primer sequences are listed in the Table 1. Gene expression changes were determined by calculating sample Ct values using relative quantification methods, normalized against the GAPDH reference gene. All reverse transcription (RT)-qPCR reactions were performed in triplicate.

**Table 1:** **Fluorescent quantitative PCR primers for related genes**

Gene	Sequence (5ʹ−3ʹ)	GenBank
GAPDH	F: GAACATCATCCCAGCGTCCA R: CGGCAGGTCAGGTCAACAAC	NM_204305.1
SPHK1	F: ACATCGCAGCCACCGTCTTTG R: CAGACATCACCACCAGCACATCC	XM_046902127.1
SGPL1	F: GCCGTCGCCTGTCACTGATG R: AACTGGCAACACCACCAAGAGAC	NM_001007946.3
S1PR1	F: TTGTTGGCTGGCGTGGCTTAC R: TGCTGCCCTCCCTTACGAACC	XM_046923371.1
S1PR2	F: GTGTACGGCGGCGACAAAGG R: GGGAGGACGGTGGAGCAGTC	XM_040653951.2
S1PR3	F: TTGTTTGTGGCTGCCTGGTGAG R: CATCCTTCGGCTTCTGGGTGTTG	XM_046936838.1
S1PR4	F: GTGTAGCCTGTCAGAACGGTGTC R: TGTCCCAGCCTGTGCCTATATCC	XM_040653638.2
ABCA1	F: CGTTCCAGCCACACTCGTCATC R: AAGAGCGAGCACAGGCAAGTTG	NM_204145.3
ABCC1	F: CACAGCACCAGAGCAGCACAG R: GCCTTTACCCTCCCAGTCTTTGC	NM_001012522.3
SPNS2	F: AATTCCTCTTGGCAGTGGCTTGG R: GATACCCGCAACGCCCAGTG	NM_001389690.2

### Caspase-1 activity assay

Caspase-1 activity in DF-1 cells was measured using the Caspase-1 Activity Assay Kit (Beyotime). After trypsin digestion, cells were centrifuged at 2000 rpm for 5 min at 4 °C. The cell culture supernatant was collected and washed once with PBS. Following the manufacturer’s instructions, the cell pellet was collected, lysed, and centrifuged at 20 000 rpm at 4 °C for 20 min. Caspase-1 activity was measured on the basis of its ability to convert the Ac-Tyr-Val-Val-Pro-Caspase-1 substrate into the active form of Caspase-1. Caspase-1 activity was measured by detecting its ability to convert acetyl-tyrosine-valine-alanine-aspartate-p-nitrophenylalanine (Ac-YVAD-pNA) to p-nitrophenylalanine (pNA).

### Histopathological examination

Histopathological examination is as follows: place the collected kidney specimens in 10% neutral formalin at room temperature, embed them in paraffin, and cut them into 5 μm slices. Stain the slices with hematoxylin and eosin (H&E) to examine pathological changes under an optical microscope.

### Western blotting

Cells were lysed in RIPA buffer containing PMSF and phosphatase inhibitors, centrifuged at 14 000 × *g* and 4 °C for 10 min, and protein concentration was determined using the BCA Protein Assay Kit (Thermo, USA). Denatured proteins were transferred to nitrocellulose membranes (GE, MA, USA) via semi-dry blotting onto 10% or 12% sodium dodecyl sulfate–polyacrylamide gel electrophoresis gels. NC membranes were blocked with 10% skim milk in TBS containing 0.05% Tween-20 (TBST) and incubated overnight at 4 °C with antibodies against S1PR1, IBV-NP, p-p38/MAPK, p38/MAPK, p-JNK/MAPK, JNK/MAPK, NLRP3, or GAPDH. The membranes were washed in TBST and incubated for 1 h with HRP-conjugated goat anti-rabbit (or anti-mouse) antibody (1:5000). After washing in TBST, the membranes were visualized using an Odyssey infrared imaging system (LI-COR Biosciences, Lincoln, NE, USA). Finally, relative protein levels were quantified using ImageJ software.

### Statistical analysis

The experimental data are in accordance with the normal distribution, and all the data are expressed as mean ± standard deviation (SD). Data were analyzed using GraphPad Prism 10 (GraphPad Software). Statistical differences between groups were analyzed using two-tailed unpaired *t*-tests for single-factor analysis or two-way analysis of variance (ANOVA) statistical tests for double factor analysis. *P*-values are denoted as follows: ns, not significant; *P < 0.05; **P < 0.01; ***P < 0.001; ****P < 0.0001. All experiments were conducted with at least three independent replicates.

## Results

### Analysis of renal metabolites following IBV infection

**Figure 1:**
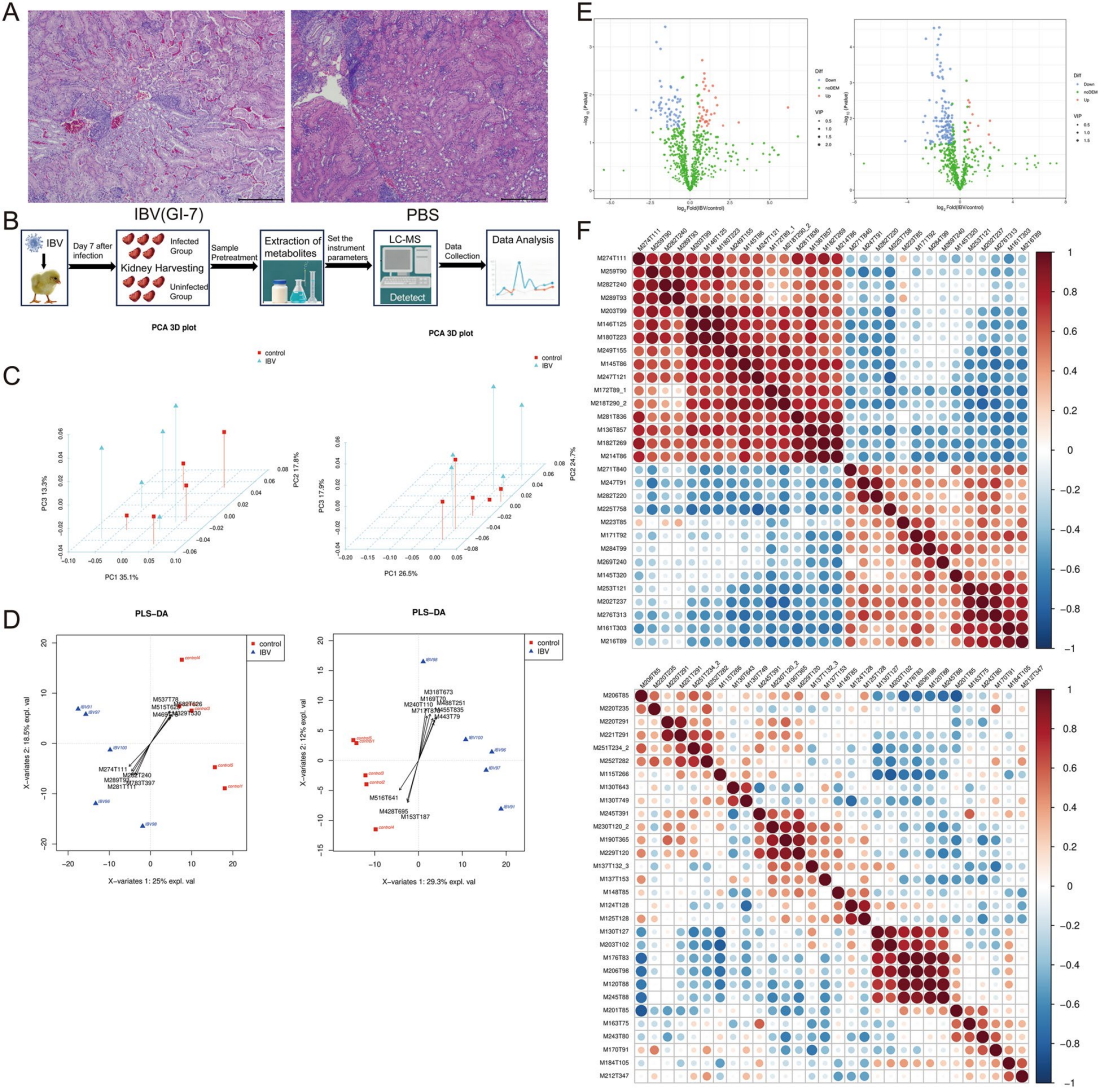
**Analysis of renal metabolites following infection with IBV strain 220198GDZC**. **A**. Histopathological examination of kidney tissue 7 days post-infection with IBV strain 220198GDZC. **B**. Complete workflow of metabolomics using liquid chromatography-mass spectrometry (LC–MS). **C**. Schematic three-dimensional (3D) visualization of principal component PCA analysis. **D**. PLS-DA model of renal metabolites between control and IBV-infected groups. **E**. Volcano plot of differentially expressed metabolites between experimental samples in the control and IBV-infected groups. **F**. Pearson correlation coefficients between metabolites, including positive and negative correlations, were calculated using the cor function in R software, with correlation relationship plots displayed.

Strain 220198GDZC, an IBV isolate obtained in our laboratory in 2021, was identified as belonging to the GI-7 lineage on the basis of S1 gene typing. A total of 20 7-day-old SPF chickens were randomly divided into 2 groups (*n* = 10 per group). One group was inoculated with 10^5^ EID₅₀ of 220198GDZC (GI-7 lineage) via intranasal routes, while the control group received PBS. Five chickens from each group were randomly euthanized 7 days post-infection, and the kidneys were collected aseptically and stored in liquid nitrogen. Histopathological analysis confirmed renal damage induced by IBV 220198GDZC infection, revealing marked tubular atrophy, detachment of tubular epithelial cells from the basement membrane, and minor epithelial necrosis (Figure 1A). Additionally, numerous bleeding points and a small amount of inflammatory cell infiltration were observed (Figure 1A). Figure 1B illustrates the complete workflow of nontargeted metabolomics using liquid chromatography-mass spectrometry (LC–MS), including sample preparation, metabolite extraction, quality control, instrument analysis, and data processing. Principal component analysis (PCA) and partial least squares discriminant analysis (PLS-DA) revealed no significant intragroup clustering or intergroup separation between the two sample groups, indicating stable peak data in the quality control samples, normal instrument response, and reliable metabolomic results suitable for downstream analysis (Figure 1C and D). The volcano plot showed that metabolites were significantly up- or downregulated in the IBV-infected group than in the control group (Figure 1E). Additionally, significant trends in correlated changes were observed for differential metabolites in the negative ion mode (Figure 1F).

### KEGG enrichment analysis of metabolic pathways

**Figure 2:**
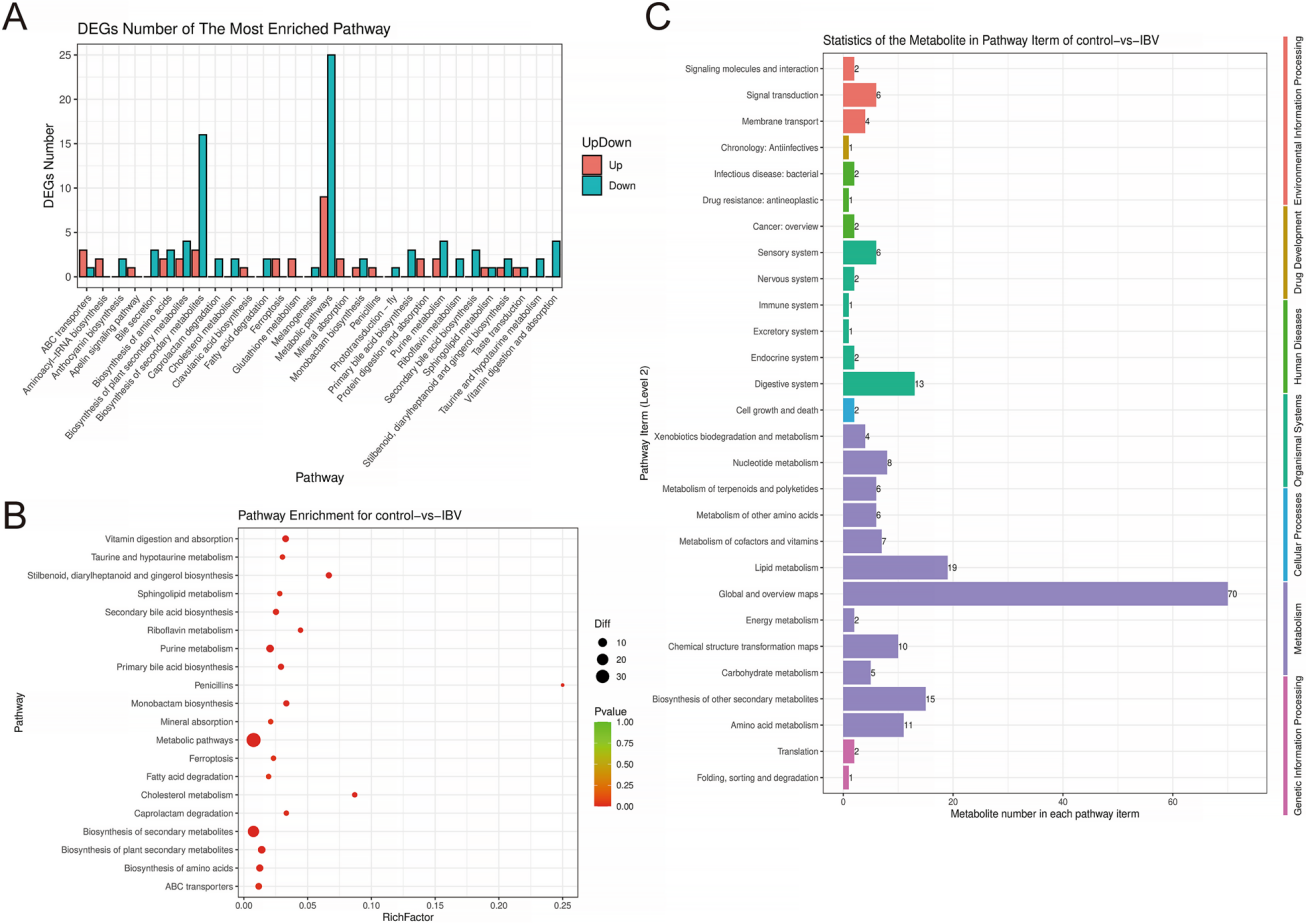
**KEGG enrichment analysis of metabolic pathways**. **A**. Bar chart showing the enrichment of up- and down-regulated metabolites in the metabolic pathways between experimental samples from the control group and the IBV-infected group. **B**. Scatter plot of enriched KEGG pathways (showing only the top 20 enriched pathway entries). **C**. Statistical results of differential metabolites contained in each entry across KEGG hierarchy levels.

Screening identified 36 upregulated and 58 downregulated anionic differential metabolites, and 12 upregulated and 134 downregulated cationic differential metabolites (Additional file 1). These findings indicate that IBV infection induces significant alterations in renal metabolites in chickens (Additional file 2). Using the KEGG Metabolome Database and MetaboAnalyzer, we retrieved the corresponding chicken pathway databases to identify signaling pathways associated with these metabolite changes. On the basis of the number of differentially expressed metabolites in each KEGG entry across all levels, the enriched pathways were classified as follows: 163 in metabolism, 25 in organismal systems, 12 in environmental information processing, 3 in genetic information processing, 5 in human diseases, 2 in cellular processes, and 1 in drug development. The results of the metabolic pathway enrichment analysis are presented in Additional files 4–6. Among the categories, metabolism showed the highest enrichment, with its secondary classifications primarily represented by global and overview maps and lipid metabolism (Figure 2C). The enrichment scatter plot indicated that significantly different metabolites were mainly enriched in general metabolic pathways and in the biosynthesis of secondary metabolites (Figure 2A and B). On the basis of the classification of differentially expressed metabolites corresponding to the KEGG-enriched pathways, one immune-related pathway was identified: Fc gamma R-mediated phagocytosis, involving the metabolite sphingosine 1-phosphate (S1P, ID: M378T777). These findings indicate that IBV infection induces significant alterations in renal lipid metabolism of chickens, with S1P potentially playing a crucial role in IBV replication.

### Changes in amino acids and energy metabolites

**Figure 3:**
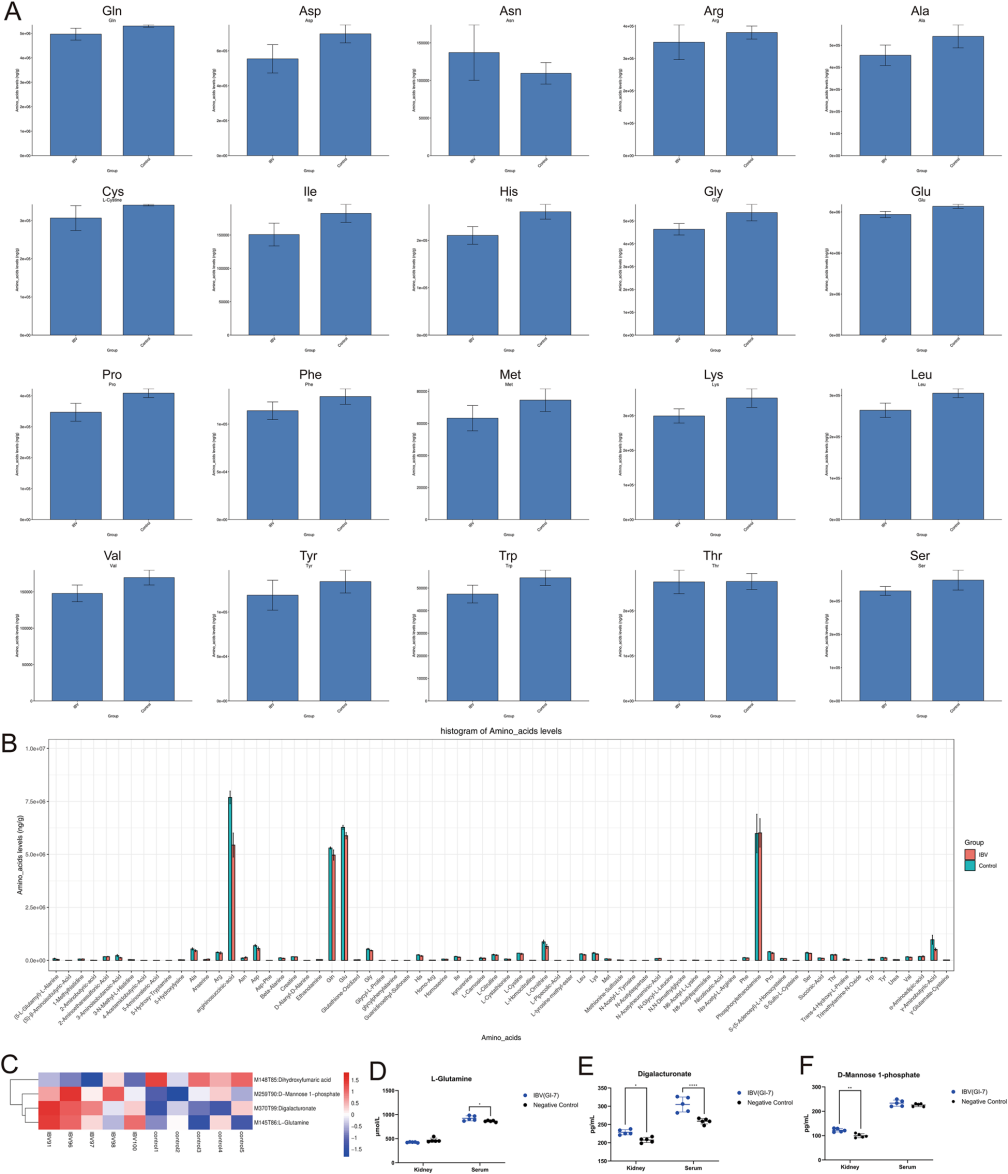
**Changes in amino acids and energy metabolites**. **A**. Bar chart showing intergroup differences in kidney metabolites between the control group and IBV-infected group. **B**. Bar chart illustrating up- and down-regulated amino acids and their derivatives. **C**. Clustered heatmap of energy metabolism pathway metabolites after normalization. **D**–**F**. ELISA-detected levels of L-glutamine, D-mannose-1-phosphate, and D-galacturonic acid in kidney and serum. Statistical differences between groups were analyzed using two-tailed unpaired *t*-tests for single-factor analysis or two-way ANOVA for double-factor analysis. *P*-values are denoted as follows: ns, not significant; *P < 0.05; **P < 0.01; ***P < 0.001; ****P < 0.0001. All experiments were conducted with at least three independent replicates.

To further investigate changes in amino acid metabolism following IBV infection, targeted quantitative measurements of kidney amino acid content were performed in SPF chickens 7 days post-infection. Metabolite ion peak areas were determined using liquid chromatography-tandem mass spectrometry (LC–MS/MS), and the measured values were compared with those of reference standards. Levels of nearly all amino acids were lower in the IBV-infected group than in the uninfected group, whereas asparagine levels were higher in the infected group (Figure 3A). Quantitative analysis of amino acid derivatives revealed that 3-aminoisobutanoic acid, argininosuccinic acid, L-ornithine, and γ-aminobutyric acid were lower in the IBV-infected group compared with the uninfected group, while other derivatives remained largely unchanged (Figure 3B). These findings suggest that IBV infection in SPF chickens enables the virus to exploit host amino acids to promote replication, resulting in reduced levels of amino acids and their derivatives.

On the basis of KEGG metabolome data analysis, four pathways related to energy metabolism were identified among all significantly enriched metabolic pathways in the differentially expressed metabolites. Among the four metabolites involved in these pathways, the significantly upregulated energy metabolites were L-glutamine, D-mannose 1-phosphate, and D-galacturonate, while one energy metabolite, d-dihydroxyfumaric acid, was significantly downregulated (Figure 3C). Levels of L-glutamine, D-mannose 1-phosphate, and D-galacturonate in the kidneys and serum following IBV infection were quantified using ELISA. The results showed that serum levels of L-glutamine and D-galacturonate were significantly higher in the infected group than in the uninfected group. In the kidneys, D-mannose 1-phosphate and D-galacturonate levels were significantly higher in the infected group compared with the uninfected group (Figure 3D–F). These findings suggest that L-glutamine, D-mannose 1-phosphate, and D-galacturonate may be involved in host energy utilization and viral replication during IBV infection.

### IBV infection induces elevated expression levels of the S1P–S1PR1 signaling axis

**Figure 4:**
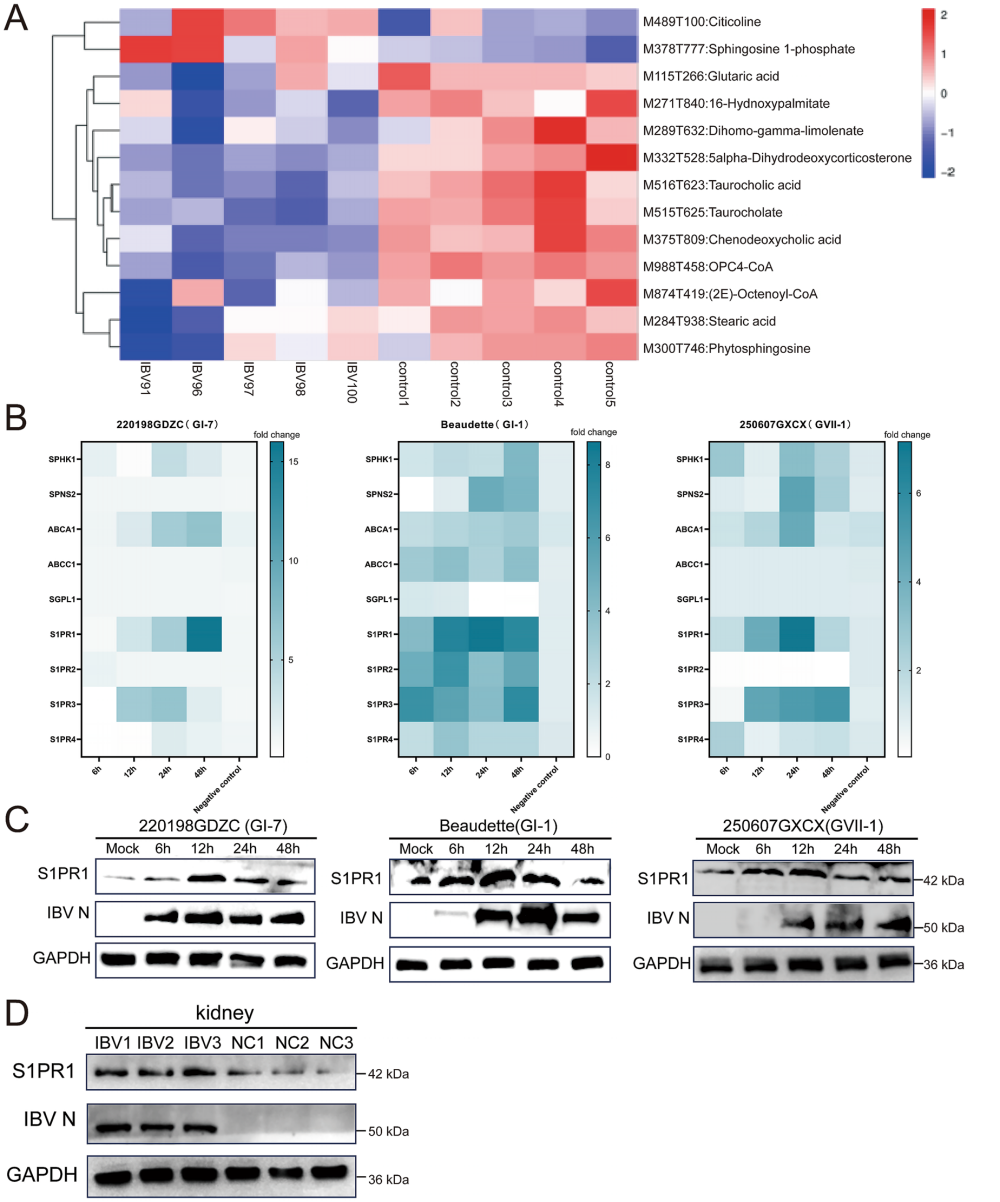
**IBV infection induces elevated S1P-S1PR1 expression levels**. **A**. Clustered heatmap of standardized quantitative data for metabolites in lipid metabolism pathways. **B**. DF-1 cells were infected with IBV (GI-1, GI-7, and GVII-1) at 1 MOI. Cells were harvested at different post-infection timepoints, and changes in S1P-related gene expression levels were detected using quantitative real-time PCR. Data were processed and heatmaps generated using GraphPad Prism 10 software. **C**. DF-1 cells were infected with IBV (GI-1, GI-7, and GVII-1) at 1 MOI. Cells were collected at different post-infection timepoints. S1PR1 protein expression was detected by western blot analysis. **D**. Kidneys were collected from SPF chickens 7 days post-infection with IBV 220198GDZC (GI-7 lineage). Protein samples (three repetitions) were extracted and analyzed by western blot to detect S1PR1 protein expression in tissues. Statistical differences between groups were analyzed using two-tailed unpaired *t*-tests for single-factor analysis or two-way ANOVA for double-factor analysis. *P*-values are denoted as follows: ns, not significant; *P < 0.05; **P < 0.01; ***P < 0.001; ****P < 0.0001. All experiments were conducted with at least three independent replicates.

KEGG analysis identified 12 metabolic pathways that were significantly enriched among the differentially expressed metabolites, including pathways related to lipid metabolism. Metabolomic analysis results demonstrated that S1P was the most markedly upregulated among all metabolites in renal tissues in response to IBV infection (Figure 4A). To further investigate S1P’s role in IBV infection, fluorescence quantitative PCR was used to detect changes in the expression of S1P-related genes at different timepoints following infection of DF-1 cells with IBV strains of different lineages (GI-1, GI-7, and GVII-1). The analyzed genes included sphingosine kinase 1 (SPHK1), sphingosine phosphate lyase 1 (SGPL1), sphingosine-1-phosphate receptor 1 (S1PR1), S1PR2, S1PR3, S1PR4, ATP-binding cassette sub-family A member 1 (ABCA1), ATP-binding cassette sub-family C member 1 (ABCC1/CFTR/MRP), and spinster homolog 2 (SPNS2). Quantitative fluorescence PCR results demonstrated a significant, time-dependent increase in S1PR1 mRNA expression following IBV infection (Figure 4B). Western blot analysis further confirmed increased S1PR1 protein expression in DF-1 cells infected with the three IBV strains, consistent with the RT-qPCR results (Figure 4C). This observation was validated in vivo: western blot analysis of SPF chicken kidneys at 7 days post-IBV infection confirmed increased S1PR1 protein expression, consistent with the in vitro findings (Figure 4D). In summary, IBV infection induced elevated expression of S1P and its receptor S1PR1, suggesting that IBV may modulate host lipid metabolism via the S1P–S1PR1 pathway.

### S1PR1 regulates the inflammatory response induced by IBV infection

**Figure 5:**
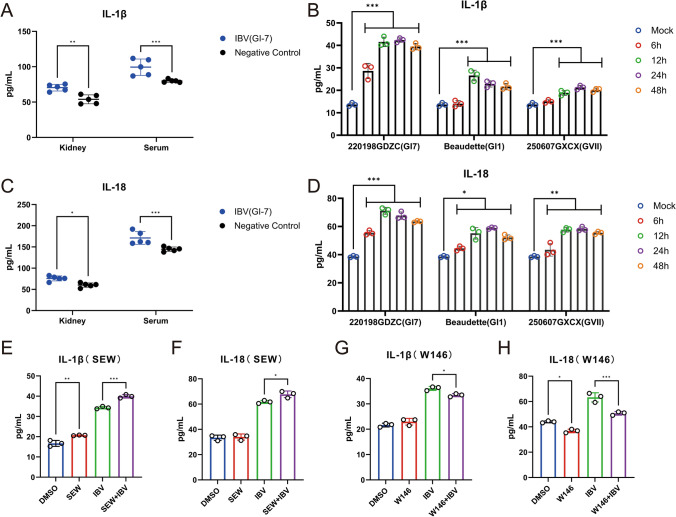
**S1PR1 regulates inflammation induced by IBV infection**. **A** and **C**. Kidneys and serum samples were collected from SPF chickens 7 days after infection with IBV 220198GDZC (GI-7 strain). IL-1β and IL-18 levels were detected by ELISA. **B** and **D**. DF-1 cells were infected with IBV (GI-1, GI-7, and GVII-1) at MOI of 1. Cell culture supernatants were collected at different post-infection timepoints and analyzed by ELISA for IL-1β and IL-18 levels. **E**–**H**. DF-1 cells were pretreated with the S1PR1-specific agonist SEW2871 (0.5 μM/mL) or the S1PR1-specific antagonist W146 (10 μM/mL), with cells inoculated with an equal volume of DMSO serving as the control. After 1 h of incubation, the original solution was removed, and the cells were infected with 1 MOI of IBV 220198GDZC (GI-7 strain). Collect cell culture supernatants 24 h post-infection and detect IL-1β and IL-18 levels using the ELISA method. Statistical differences between groups were analyzed using two-tailed unpaired *t*-tests for single-factor analyses or two-way ANOVA for double-factor analyses. *P*-values are denoted as follows: ns, not significant; *P < 0.05; **P < 0.01; ***P < 0.001; ****P < 0.0001. All experiments were conducted with at least three independent replicates.

Research has indicated that S1PR1 is closely associated with inflammatory responses during viral infections and various diseases and has been identified as a key target for antiviral drug development and clinical treatment [20–23]. To investigate the association between IBV-induced inflammation and S1PR1, we first measured the levels of host-produced inflammatory cytokines (IL-18 and IL-1β) induced by different IBV strains both in vivo and in vitro as indicators of inflammatory activation. One-day-old SPF chickens were infected with IBV strains (GI-7 lineage). Kidneys and serum were collected 7 days post-infection, and cytokine levels were measured using ELISA. The results showed significantly higher levels of IL-18 and IL-1β in both kidneys and serum compared with the negative control group (Figure 5A and C). DF-1 cells were infected with three IBV strains (GI-1, GI-7, and GVII-1), and cell supernatants were collected at different timepoints for cytokine detection. The results showed that all three IBV strains induced significant increases in IL-18 and IL-1β at 12 h post-infection, indicating that IBV infection markedly induces inflammatory responses (Figure 5B and D). Subsequently, we focused on the role of S1PR1 (drug concentrations of SEW2871 and W146 did not affect cell viability; Additional file 1). Cells were pretreated with SEW2871, a specific activator of S1PR1, followed by IBV infection, and IL-18 and IL-1β secretion levels in supernatants were measured. Results showed that IL-18 (68.38 pg/mL) and IL-1β (40.82 pg/mL) levels in the SEW2871-treated, IBV-infected group were significantly higher than those in the untreated, IBV-infected group (Figure 5E and F). Using the same approach, cells were pretreated with the S1PR1 antagonist W146, and supernatants were collected 24 h after IBV infection. In contrast, IL-18 (51.06 pg/mL) and IL-1β (33.51 pg/mL) levels in the W146-treated, IBV-infected group were significantly lower than those in the untreated, IBV-infected group (Figure 5G and H). These findings indicate that IBV infection significantly activates the host inflammatory response, whereas S1PR1 modulates this IBV-mediated inflammation.

### IBV activates the p38/MAPK pathway via S1PR1

**Figure 6:**
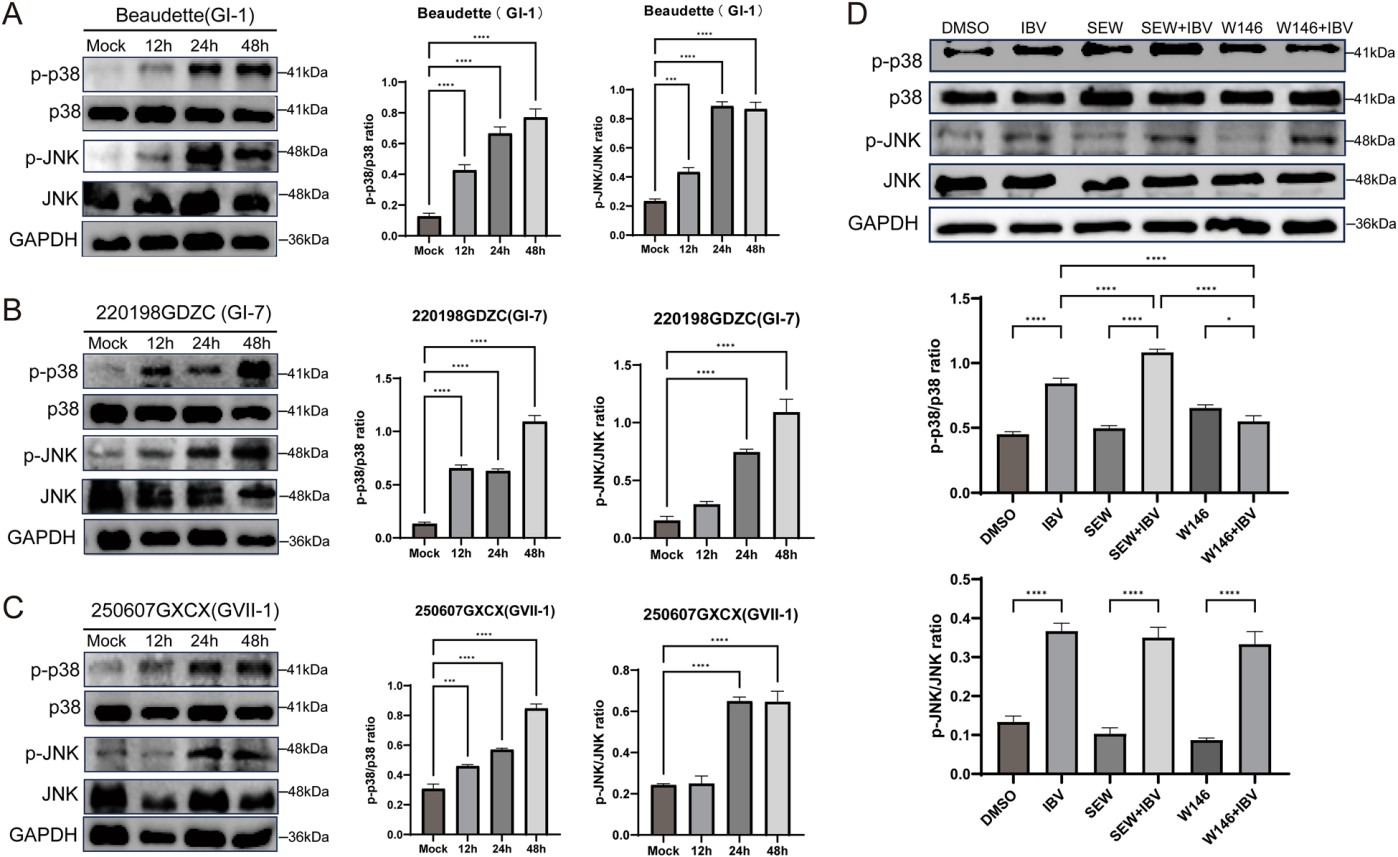
**IBV activates the p38/MARK pathway via S1PR1**. **A**–**C**. DF-1 cells were infected with IBV (GI-1, GI-7, and GVII-1) at MOI of 1. Cells were harvested at different post-infection timepoints. Western blot analysis detected the protein expression of p38/p-p38 and JNK/p-JNK in the cells. **D**. DF-1 cells were pretreated with the S1PR1-specific agonist SEW2871 (0.5 μM/mL) or the S1PR1-specific antagonist W146 (10 μM/mL), with cells inoculated with an equal volume of DMSO serving as the control. After incubation for 1 h, remove the original solution and infect cells with 1 MOI of IBV 220198GDZC (GI-7 strain). Collect cells 24 h post-infection and detect the protein expression of p38/p-p38 and JNK/p-JNK in the cells via western blot. Use ImageJ software for quantitative analysis of protein gray values. Statistical differences between groups were analyzed using two-tailed unpaired *t*-tests for single-factor analysis or two-way ANOVA for double factor analysis. *P*-values are denoted as follows: ns, not significant; *P < 0.05; **P < 0.01; ***P < 0.001; ****P < 0.0001. All experiments were conducted with at least three independent replicates.

To further examine the signaling pathways through which S1PR1 regulates IBV-mediated inflammation, we investigated MAPK pathway activation during infection. Previous studies have shown that viral infection can activate the MAPK signaling pathway—particularly the p38/MAPK cascade—to induce physiological or pathological effects, and that MAPK signaling plays a key role in mediating inflammatory responses in various infections or diseases [24–28]. Therefore, we assessed the relationship between S1PR1 and the p38 and JNK/MAPK signaling pathways during IBV infection. DF-1 cells were infected with three IBV strains (GI-1, GI-7, and GVII-1). Cells were collected at different timepoints post-infection, and the phosphorylation levels of JNK/MAPK and p38/MAPK were evaluated by western blot analysis. The results showed that the phosphorylation levels of the JNK/MAPK and p38/MAPK signaling pathways increased in a time-dependent manner following infection with all three IBV strains, with a significant elevation observed at 24 h post-infection. These findings indicated that IBV infection activated both the JNK/MAPK and p38/MAPK pathways (Figure 6A–C). The cells were subsequently pretreated with the specific S1PR1 activator, SEW2871, or the S1PR1 inhibitor W146, followed by IBV infection. Cells were collected at 24 h post-infection to assess the phosphorylation levels of the JNK/MAPK and p38/MAPK signaling pathways. The results showed elevated phosphorylation of the p38/MAPK pathway following IBV infection. SEW2871 treatment further increased p38/MAPK phosphorylation, whereas W146 treatment markedly reduced it (Figure 6D). In contrast, phosphorylation levels in the JNK/MAPK pathway remained unchanged after treatment with either SEW2871 or W146 (Figure 6D). Overall, these findings indicate that IBV infection activates both the JNK/MAPK and p38/MAPK signaling pathways, whereas S1PR1 specifically modulates IBV-induced activation of the p38/MAPK pathway without affecting JNK/MAPK signaling.

### IBV regulates the NLRP3/caspase-1 inflammasome via S1PR1

**Figure 7:**
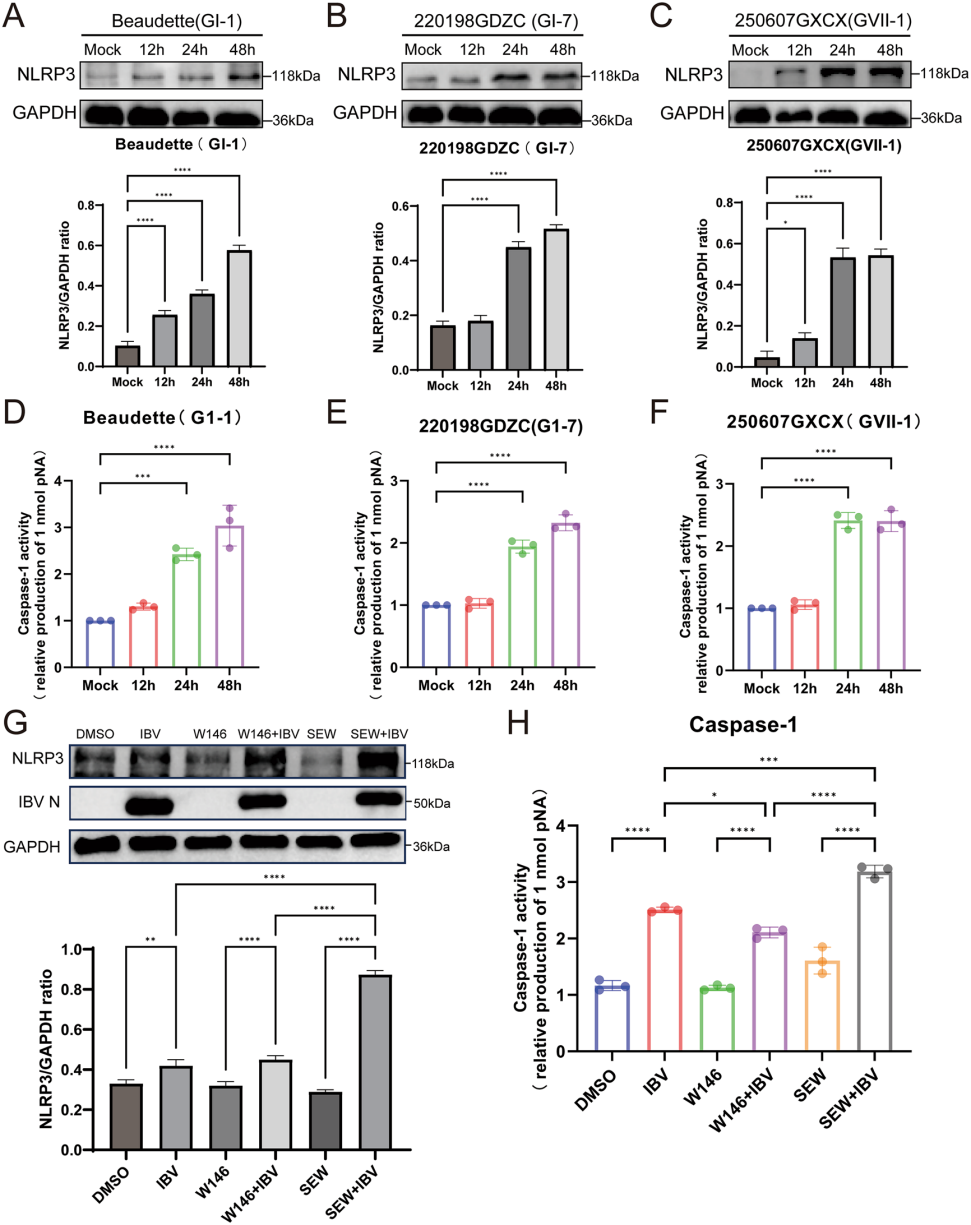
**IBV regulates NLRP3/Caspase-1 inflammasome via S1PR1**. **A**–**C**. DF-1 cells were infected with IBV (GI-1, GI-7, and GVII-1) at 1 MOI. Cells were harvested at different post-infection timepoints. NLRP3 protein expression was detected by western blot. **D**–**F**. DF-1 cells were infected with IBV (GI-1, GI-7, and GVII-1) at MOI of 1. Cells were harvested at different post-infection timepoints. Caspase-1 activity was measured using a colorimetric assay kit, with activity calculated on the basis of standard curve values. **G** and **H**. DF-1 cells were pretreated with the S1PR1-specific agonist SEW2871 (0.5 μM/mL) or the S1PR1-specific antagonist W146 (10 μM/mL). Cells treated with an equal volume of DMSO served as the control. After 1 h incubation, the original solution was removed, and cells were infected with 1 MOI of IBV 220198GDZC (GI-7 strain). At 24 h post-infection, cells were harvested to detect NLRP3 protein expression (G) or caspase-1 activity (H) via western blot analysis. ImageJ software was used for quantitative analysis of protein grayscale values. Statistical differences between groups were analyzed using two-tailed unpaired *t*-tests for single-factor analysis or two-way ANOVA for double-factor analysis. *P*-values are denoted as follows: ns, not significant; *P < 0.05; **P < 0.01; ***P < 0.001; ****P < 0.0001. All experiments were conducted with at least three independent replicates.

NLRP3/Caspase-1 is an essential upstream component required for the production of the inflammatory cytokines IL-18 and IL-1β [29]. Therefore, we investigated the effects of S1PR1 and the NLRP3/Caspase-1 during IBV infection. DF-1 cells were infected with three IBV strains (GI-1, GI-7, and GVII-1). Cells were harvested at different timepoints post-infection, and NLRP3 protein expression levels were assessed by western blot analysis. The results demonstrated a significant increase in NLRP3 protein expression following IBV infection (Figure 7A–C). Using the same sample preparation approach, we also evaluated changes in Caspase-1 activity after IBV infection. The results showed a significant increase in Caspase-1 activity at 24 h post-infection (Figure 7D–F). These findings indicate that IBV infection activates the NLRP3/Caspase-1 inflammasome. Subsequently, the cells were pretreated with the specific S1PR1 agonist SEW2871 or the antagonist W146, followed by IBV infection. The cells were harvested 24 h post-infection to assess NLRP3 protein expression and Caspase-1 activity. Our results showed that NLRP3 protein expression increased following IBV infection. Treatment with SEW2871 significantly enhanced NLRP3 protein expression, whereas W146 treatment did not affect NLRP3 expression (Figure 7G). Caspase-1 activity assays revealed a consistent trend: SEW2871 treatment significantly enhanced Caspase-1 activity, whereas W146 treatment markedly reduced it (Figure 7H). These findings indicate that IBV modulates NLRP3/Caspase-1 inflammasome activation via S1PR1.

### Knockdown of S1PR1 significantly suppresses IBV-induced inflammation

**Figure 8:**
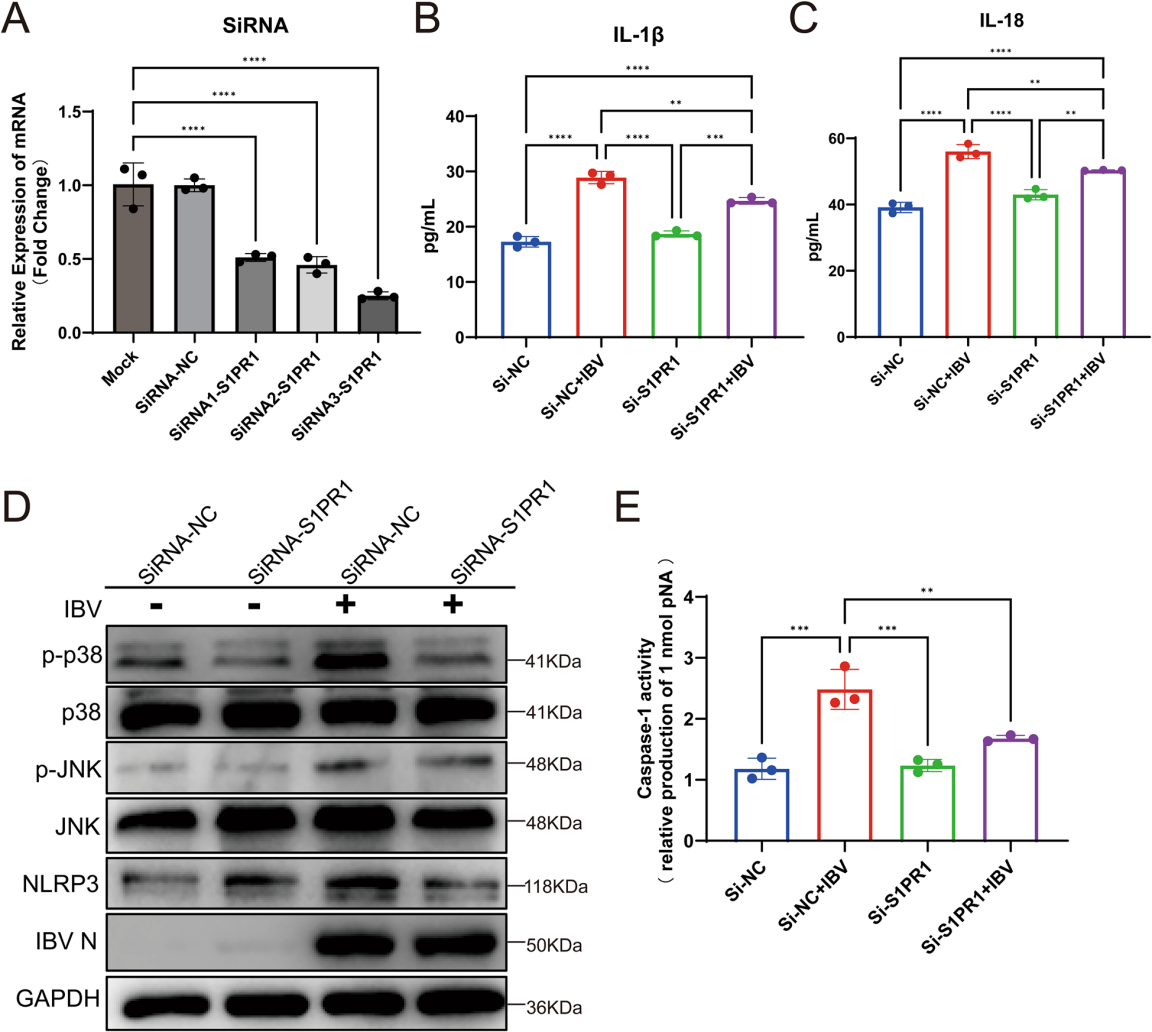
**Knockdown of S1PR1 significantly suppresses IBV-induced inflammation**. **A**. Detection of siRNA knockdown efficiency. Three pairs of siRNA were transfected into DF-1 cells. S1PR1 expression levels were assessed by RT-qPCR 24 h post-transfection. **B** and **C**. Effects of S1PR1 knockdown on IL-1β and IL-18. At 24 h after siRNA transfection into DF-1 cells, cells were infected with IBV 220198GDZC (GI-7 strain) at 1 MOI. Cell supernatants were collected 24 h post-infection. Inflammatory cytokine levels were measured by ELISA. **D**. Effects of S1PR1 knockdown on related signaling pathways. Cells were treated identically, and western blot was used to detect phosphorylation levels of p—38/MAPK and JNK/MAPK, as well as NLRP3 expression. **E**. Effects of S1PR1 knockdown on caspase-1. Cells were treated identically, and caspase-1 activity was measured using an assay kit. Statistical differences between groups were analyzed using two-tailed unpaired *t*-tests for single-factor analysis or two-way ANOVA for double-factor analysis. *P*-values are denoted as follows: ns, not significant; *P < 0.05; **P < 0.01; ***P < 0.001; ****P < 0.0001. All experiments were conducted with at least three independent replicates.

To investigate the effects of gene-level intervention on S1PR1 in mediating inflammation during IBV infection, we designed three pairs of siRNAs targeting the chicken S1PR1 gene to knock down its transcriptional expression. These siRNAs were transfected into DF-1 cells, and after 24 h, the cells were harvested. Fluorescent quantitative PCR was used to detect S1PR1 gene expression levels. Results showed that all three siRNA pairs significantly suppressed S1PR1 expression, with siRNA3 exhibiting the highest knockdown efficiency (Figure 8A). Subsequent experiments employed siRNA3 for gene knockdown. The effects of S1PR1 knockdown on IBV-mediated inflammation were then investigated. DF-1 cells were infected with IBV 24 h after S1PR1 knockdown, and IL-1β and IL-18 levels in cell supernatants were measured 24 h post-infection. Results showed that the Si-NC group exhibited significantly elevated levels of inflammatory cytokines following IBV infection, whereas IBV-induced cytokine levels were suppressed after S1PR1 knockdown (Figure 8B–C). Further investigation explored the effects of S1PR1 knockdown on related signaling pathways. Western blot analysis revealed that IBV infection activated p38/MAPK, JNK/MAPK, and NLRP3 (Figure 8D). Knocking down S1PR1 significantly reduced the phosphorylation level of p38/MAPK and the expression of NLRP3, without affecting the phosphorylation level of JNK/MAPK (Figure 8D). Furthermore, S1PR1 knockdown significantly inhibited IBV infection-mediated caspase-1 activation (Figure 8E). Collectively, these findings indicate that S1PR1 knockdown suppresses p38/MAPK and NLRP3/caspase-1 activation, thereby attenuating IBV infection-induced inflammatory responses.

## Discussion

IB is a viral disease that affects poultry and causes substantial economic losses to the global poultry industry. Following IBV infection, chickens exhibit a range of symptoms primarily affecting the respiratory tract, with severe pathological changes particularly evident in the kidneys. As a vital organ for metabolism, the kidneys are commonly affected during IBV infection, showing enlargement, hemorrhage, urate deposition, and even “mottled kidneys” [30]. However, it remains unclear how IBV hijacks amino acids, energy, or lipid molecules within the renal metabolic pathways. These metabolic alterations reflect biochemical interactions between host defense mechanisms and viral invasion during the course of viral infection [31].

In recent years, metabolomics has been widely applied in poultry viral infection research to elucidate host responses during infection. Following Newcastle disease virus (NDV) infection, 305 metabolites were significantly altered. Increased levels of amino acids and nucleotides may facilitate viral protein synthesis and genomic amplification, thereby promoting NDV infection [32]. In the bursa of Fabricius, 12 metabolites were identified as potential biomarkers of renal-type IBV infection, with strong correlations observed between these metabolic biomarkers and inflammatory cytokines [15]. Investigating the mechanisms by which viruses regulate host metabolism and developing preventive or therapeutic strategies targeting altered metabolic pathways represent critical approaches for antiviral intervention. In this study, we employed LC–MS/MS metabolomics to analyze differential metabolites in the kidneys of SPF chickens infected with IBV, aiming to elucidate changes in amino acid, energy, and lipid metabolism following infection. Our findings indicate that S1PR1 plays a crucial role in IBV-mediated inflammatory responses, providing new insights into the interactions between IBV and host metabolism.

We conducted a detailed assessment of altered metabolites and metabolic pathways in the kidneys following IBV infection. The significantly altered metabolites primarily included amino acids and their derivatives, as well as lipid metabolites. Amino acids are essential cellular components that serve as raw materials for synthesizing biomolecules such as proteins, peptides, and nucleic acids, while also providing precursors for other metabolic pathways. Viral infections can hijack amino acids, disrupting normal metabolic pathway functions. The pseudorabies virus (PRV) reprograms metabolic activity in PK-15 cells, with glycolysis, the pentose phosphate pathway, and glutamine metabolism for nucleotide biosynthesis serving as critical pathways for enhanced PRV replication [33]. Studies have shown that exogenous supplementation of cysteine, methionine, lysine, and nucleosides in IBDV-infected cells results in a 20.7-fold increase in viral titers [34]. During the early stages of ASFV infection, numerous amino acids are significantly upregulated; however, in the later stages, cellular amino acids are depleted, and aspartic and glutamic acids promote ASFV replication [35]. In this study, LC–MS/MS was used to quantitatively analyze amino acids following IBV infection. Most amino acids and their derivatives exhibited a decreasing trend post-infection, likely reflecting the timing of kidney sample collection on day 7 post-infection—a late stage of viral infection during which the virus hijacks amino acids to facilitate replication. Additionally, ELISA revealed increased levels of L-glutamine, D-mannose 1-phosphate, and D-galacturonate following IBV infection, suggesting that these compounds play crucial roles in viral replication.

Proteomic studies of kidneys after IBV infection have revealed that metabolic pathways respond during the early phase (3 dpi), whereas immune-related signaling pathways respond during the mid-to-late phase (5 and 7 dpi). Among these, the type I interferon (IFN-I) signaling pathway is a key component of the host response to IBV infection [36]. In this study, KEGG enrichment analysis identified metabolic pathways affected by IBV infection, including an important immune-related pathway, Fc gamma R-mediated phagocytosis, in which S1P exhibited significant changes. S1P is a biologically active sphingosine derived from sphingosine kinases (SphKs), and primarily exerts physiological and pathological effects by activating G protein-coupled cell surface receptors, specifically the endothelial differentiation gene family known as S1P receptors (S1PR1-5) [37]. Viruses can manipulate one or more molecules within the S1P family to regulate immune signaling pathways, thereby promoting their own replication. For example, the NSI protein of avian influenza virus reduces the innate immune response to IFN-Is by inducing ubiquitination and inhibiting S1P lyase [38]. S1P signaling also contributes to virus-mediated shedding of epithelial cells, as inhibition of S1P signaling delays the shedding of infected cells and increases viral titers in epithelial cells [39]. These findings indicate that the S1P family plays a crucial role in viral infection. Therefore, we investigated the role of S1P in IBV infection. The S1P family primarily comprises precursor, transport, and receptor proteins, with key genes including SPHK1, SGPL1, SPNS2, ABCA1, ABCC1, and S1PR1-4 [40]. Fluorescent quantitative PCR revealed significant upregulation of S1PR1 following infection with different IBV lineages, with the most pronounced increase observed after infection with the cell-adapted Beaudette strain. Additionally, S1PR3 exhibits an upward trend after infection, although its specific regulatory mechanisms remain to be elucidated. Subsequent in vivo and in vitro validations confirmed increased expression of S1PR1 in both cells and kidneys following IBV infection, indicating that IBV enhances the expression of the S1P–S1PR1 signaling axis.

S1PR1 has been demonstrated to be a key regulator of cytokine storms and inflammatory responses, serving as a crucial target for modulating immune cell migration and mitigating inflammatory reactions [41–43]. This study confirms the involvement of S1PR1 in IBV infection. Therefore, we further investigated the link between IBV infection and S1PR1-mediated regulation of inflammatory responses. Previous research has shown that IBV promotes the release of inflammatory cytokines via the TLR7/NF-κB signaling pathway, thereby contributing to kidney damage [44]. In this study, infection with IBV at 7 days post-infection (dpi) significantly increased IL-1β and IL-18 levels in both kidney tissue and serum. Similarly, infection with three IBV strains of different lineages caused a significant increase in IL-1β and IL-18 levels in cells at 12 h post-infection (hpi), indicating that IBV infection activates inflammatory responses both in vivo and in vitro. To further determine the role of S1PR1 in IBV-induced inflammatory responses, cells were treated with the S1PR1-specific agonist SEW2871, which significantly increased IBV-induced IL-1β and IL-18 secretion. Conversely, treatment with the S1PR1-specific inhibitor W146 markedly reduced IL-1β and IL-18 secretion. These findings demonstrate that S1PR1 regulates IBV-induced inflammatory cytokine expression. The therapeutic potential of S1P signaling pathways may also extend to the treatment of other immune-mediated and inflammatory diseases [45]. Therapeutic drugs targeting SphK or S1PRs may aid in developing strategies to prevent the spread of HIV-1 within immune cells [46]. The results of this study demonstrated the role of S1PR1 in IBV-mediated inflammatory responses, establishing S1PR1 as a key target for alleviating IBV-induced clinical symptoms.

S1PR1 regulates the migration of immune-inflammatory cells and the expression of inflammatory cytokines through multiple pathways. Novel sphingolipids targeting S1PR1 enhance S1PR1/Akt/mTOR signaling, thereby attenuating neuroinflammation [47]. The traditional Chinese medicine Qing Bi Yin (QBY) inhibits the proliferation and differentiation of Th17 cells and modulates the phosphorylation of STAT3 and SMAD2 via the S1P/S1PR1 pathway, reducing inflammatory cytokine secretion by Th17 cells [48]. In viral infections, NDV infection promotes S1PR1 expression via NLRP3/Caspase-1 inflammasome activation, inducing IL-1β expression [49]. Additionally, human immunodeficiency virus (HIV) replication in endothelial cells depends on SphK activity and the S1PR1, involving activation of phosphatidylinositol-3-kinase and the small GTPase Ras-related C3 botulinum toxin substrate 1[50]. In this study, we investigated the relationship between S1PR1 and the MAPK signaling pathway following IBV infection. Treatment with the specific activator SEW2871 significantly increased IBV-induced p38/MAPK phosphorylation, whereas treatment with the specific inhibitor W146 significantly reduced it. Consistent with findings in NDV, our results indicated that modulation of S1PR1 did not significantly affect IBV-induced JNK/MAPK phosphorylation levels [49]. These findings suggest that S1PR1 plays distinct roles during viral infections: it promotes inflammatory responses and exacerbates inflammatory damage, while modulating immune response to influence immune evasion.

To further elucidate the mechanism by which S1PR1 modulates inflammatory cytokine expression, we examined the effects of SEW2871 and W146 on NLRP3/Caspase-1 inflammasome activation. Activation of S1PR1 by SEW2871 significantly increased NLRP3 expression and Caspase-1 activity, whereas S1PR1 inhibition markedly reduced Caspase-1 activity. These results suggest that S1PR1 regulates IBV-induced IL-1β and IL-18 expression via the NLRP3/Caspase-1 inflammasome pathway. Knockdown of S1PR1 using siRNA yielded identical results. Notably, modulation of S1PR1 did not affect viral protein expression, indicating that its regulatory role occurs solely at the host defense level without influencing viral replication.

**Figure 9:**
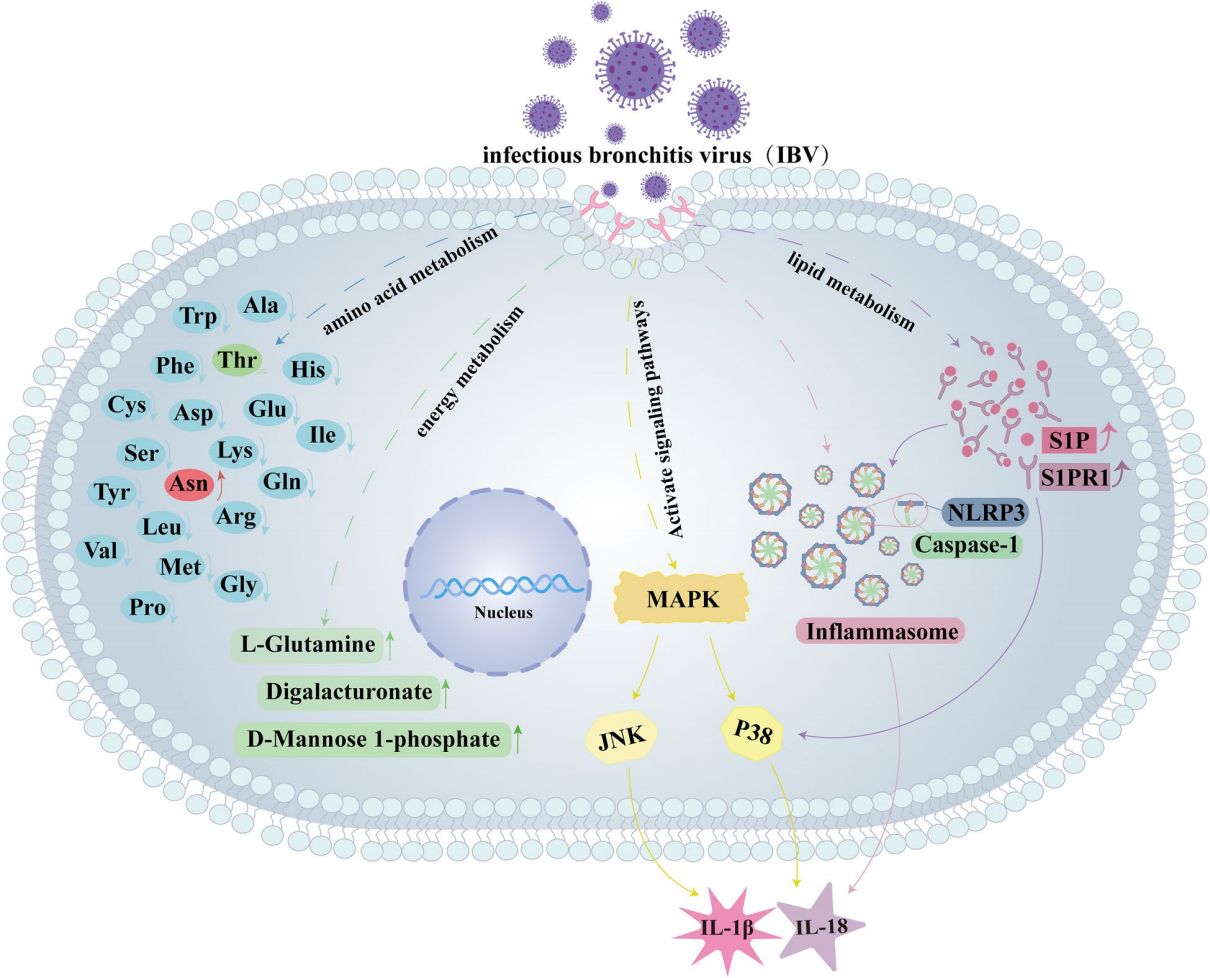
**Schematic diagram of metabolic changes induced by IBV infection and IBV regulation of inflammatory responses via S1PR1**. IBV infection causes significant alterations in amino acid and energy metabolism while regulating the p38/MAPK signaling pathway and NLRP3/Caspase-1 inflammasome through S1PR1, thereby inducing the secretion of inflammatory cytokines IL-1β and IL-18.

MAPK pathway serves as one of the central signaling axes for virus-induced host inflammatory responses. Following IBV infection, both p38/MAPK and JNK/MAPK are activated to mediate downstream physiological effects. In this study, we discovered that S1PR1 regulates inflammatory responses via p38/MAPK, while JNK/MAPK does not participate in this process. JNK/MAPK may be regulated by other host factors or viral proteins to mediate viral replication, cellular damage, and stress responses. This indicates that S1PR1 may possess specific target sites to mediate inflammatory responses following IBV infection. The NLRP3/caspase-1 inflammasome, a precursor of inflammatory responses, is also regulated by S1PR1 following IBV infection. The occurrence of inflammation is modulated by multiple host factors and pathways. Our study demonstrates that the S1P-S1PR1 axis is upregulated after IBV infection and regulates inflammatory responses via p38/MAPK and NLRP3/caspase-1 pathways. Additionally, other pathways such as PI3K/AKT or NFκB may participate in this process, the process of viral infection-induced inflammation involves a cascade reaction among multiple host factors, and additional targets warrant exploration. In summary, IBV infection enhances the S1P–S1PR1 axis while simultaneously activating the p38 and JNK/MAPK signaling pathways and the NLRP3/Caspase-1 inflammasome. Furthermore, S1PR1 modulates p38/MAPK signaling and NLRP3/Caspase-1 inflammasome activity, thereby mediating IL-1β and IL-18 expression (Figure 9). In addition to inflammation, kidney damage caused by IBV infection may also be associated with endoplasmic reticulum stress and apoptosis [51]. The relationship between S1PR1 and its associated pathways requires further investigation.

The latest research demonstrated through in vivo and in vitro experiments that IBV infection activates the NLRP3 inflammasome, thereby promoting the maturation and secretion of IL-1β (interleukin-1β). Furthermore, it was found that NLRP3 is primarily localized within Aquaporin-2 (AQP2)-positive epithelial cells, making it a key target for IBV-induced nephropathy [52]. In this study, we similarly observed that IBV infection activates the NLRP3/caspase-1 inflammasome, thereby inducing host inflammatory responses. However, unlike previous studies, our research innovatively identified S1PR1 as a regulator of post-IBV infection inflammation on the basis of host metabolic responses. Consequently, we further focused on the relationship between S1PR1 and inflammatory pathways, exploring how S1PR1 modulates IBV-induced inflammation through inflammatory targets within the host immune metabolism. In summary, previous studies primarily emphasized the pathogenesis of kidney disease through NLRP3 activation by IBV infection, whereas our research concentrates more on S1PR1 within metabolic pathways and its role in inflammatory injury. We employed metabolomics to systematically analyze IBV-induced alterations in amino acid, energy, and lipid metabolism and identified S1PR1 as a key regulator of IBV-mediated inflammatory responses. However, the precise roles of differentially expressed genes involved in amino acid and energy metabolism—as well as their viral replication and host metabolic pathways such as the tricarboxylic acid cycle, pentose phosphate pathway, and glycolysis—warrant further investigation. Beyond S1PR1, other S1P-related molecules, including SPHK1, SPNS2, and S1PR3, may provide additional insights into viral pathogenic mechanisms and immune evasion. Overall, our study highlights the role of S1PR1 in IBV infection and suggests that S1PR1 may serve as a potential therapeutic target for IB, supporting the development of novel prevention and control strategies that target host metabolic pathways.

## Supplementary Information

**Additional file 1: Principal component analysis of renal metabolites.** A. PCA analysis of renal metabolites. B. Cluster analysis heatmap of standardized quantitative metabolite data. C. Statistics on the number of differentially metabolized compounds.

**Additional file 2: Statistical Analysis of Differentially Expressed Metabolites in Kidneys**. A. Cluster analysis and heatmap visualization of normalized intergroup metabolite data. B. Z-score analysis was performed on differential metabolites and visualized. Z-score calculation is based on the mean and standard deviation of the data, expressed by the formula: z = (x – μ) / σ. Here, x represents a specific score, μ denotes the mean, and σ indicates the standard deviation.

**Additional file 3: Effects of S1PR1-specific agonist SEW2871 and antagonist W146 on DF-1 cell activity.** DF-1 cells were seeded at a density of 5 × 10³ cells per well in a 96-well plate. After treatment with W146, SEW2871, or dimethyl sulfoxide (DMSO), 10 μl of CCK-8 reagent was added and incubated at 37°C for 2 hours. Absorbance was measured at a reference wavelength of 450 nm.

**Additional file 4: Enrichment results for Amino acid metabolism pathways.**

**Additional file 5: Enrichment results for carbohydrate metabolism pathways.**

**Additional file 6: Enrichment results for lipid metabolism pathways.**

## Data Availability

The datasets used and/or analyzed during the current study are available from the corresponding author on reasonable request.
