# Supplementary material for: Avian coronaviruses induce inflammatory responses by activating p38/MAPK signaling and NLRP3/caspase-1 inflammasomes through sphingosine-1-phosphate receptor 1
Source: Vet Res. 2026 May 23;57:83. doi: 10.1186/s13567-026-01768-0 (PMC13198749; doi:10.1186/s13567-026-01768-0)
Supplement: Supplementary file 1 — Additional file 1: Principal component analysis of renal metabolites. A. PCA analysis of renal metabolites. B. Cluster analysis heatmap of standardized quantitative metabolite data. C. Statistics on the number of differentially metabolized compounds. [file 13567_2026_1768_MOESM1_ESM.docx]

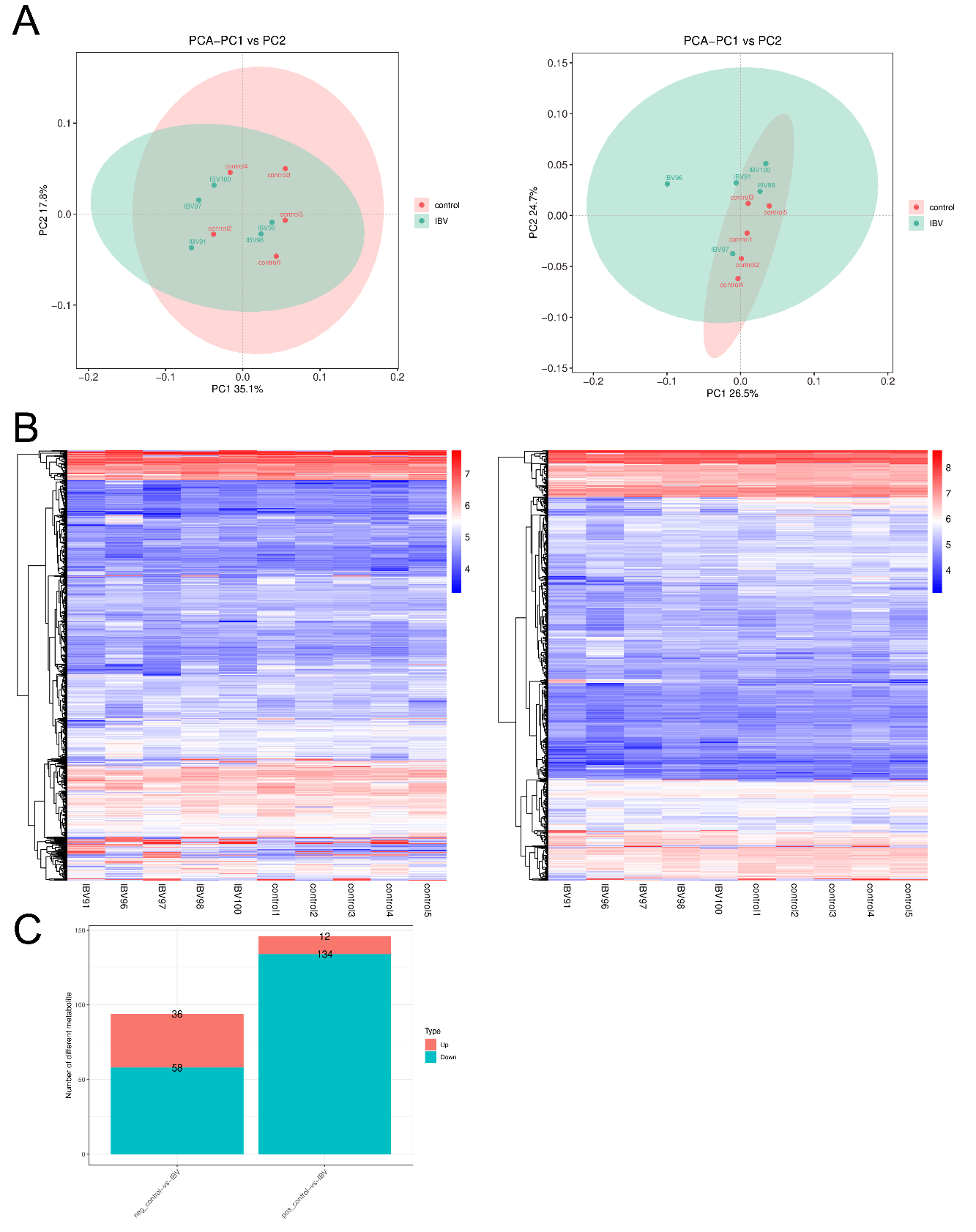


**Additional file 1.** Principal component analysis of renal metabolites. A. PCA analysis of renal metabolites. B. Cluster analysis heatmap of standardized quantitative metabolite data. C. Statistics on the number of differentially metabolized compounds.
