# Supplementary material for: Avian coronaviruses induce inflammatory responses by activating p38/MAPK signaling and NLRP3/caspase-1 inflammasomes through sphingosine-1-phosphate receptor 1
Source: Vet Res. 2026 May 23;57:83. doi: 10.1186/s13567-026-01768-0 (PMC13198749; doi:10.1186/s13567-026-01768-0)
Supplement: Supplementary file 2 — Additional file 2: Statistical Analysis of Differentially Expressed Metabolites in Kidneys. A. Cluster analysis and heatmap visualization of normalized intergroup metabolite data. B. Z-score analysis was performed on differential metabolites and visualized. Z-score calculation is based on the mean and standard deviation of the data, expressed by the formula: z = (x – μ) / σ. Here, x represents a specific score, μ denotes the mean, and σ indicates the standard deviation. [file 13567_2026_1768_MOESM2_ESM.docx]

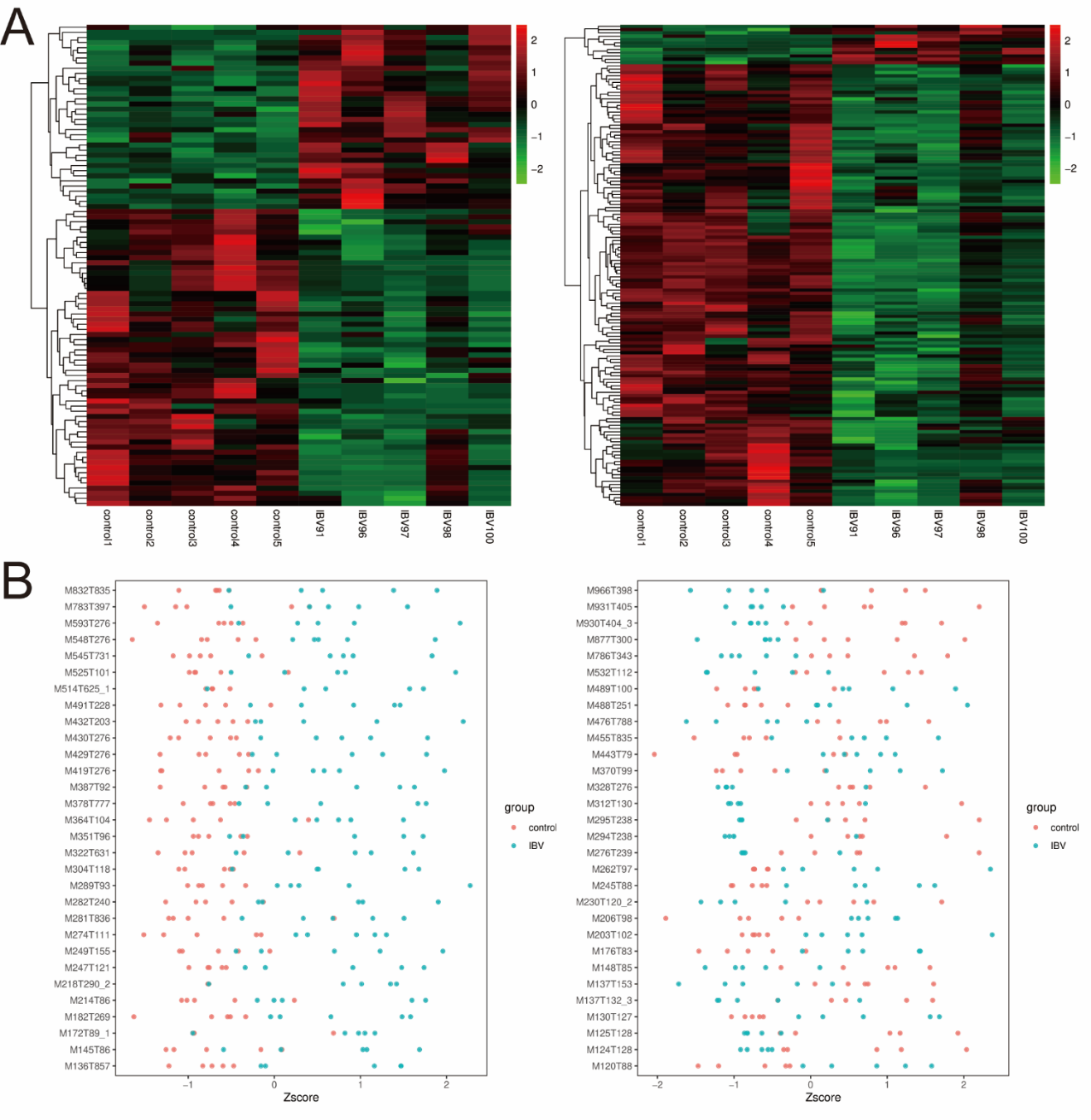


**Additional file 2.** Statistical Analysis of Differentially Expressed Metabolites in Kidneys. A. Cluster analysis and heatmap visualization of normalized intergroup metabolite data. B. Z-score analysis was performed on differential metabolites and visualized. Z-score calculation is based on the mean and standard deviation of the data, expressed by the formula: z = (x – μ) / σ. Here, x represents a specific score, μ denotes the mean, and σ indicates the standard deviation.
