# Supplementary material for: Avian coronaviruses induce inflammatory responses by activating p38/MAPK signaling and NLRP3/caspase-1 inflammasomes through sphingosine-1-phosphate receptor 1
Source: Vet Res. 2026 May 23;57:83. doi: 10.1186/s13567-026-01768-0 (PMC13198749; doi:10.1186/s13567-026-01768-0)
Supplement: Supplementary file 3 — Additional file 3: Effects of S1PR1-specific agonist SEW2871 and antagonist W146 on DF-1 cell activity. DF-1 cells were seeded at a density of 5 × 10³ cells per well in a 96-well plate. After treatment with W146, SEW2871, or dimethyl sulfoxide (DMSO), 10 μl of CCK-8 reagent was added and incubated at 37°C for 2 hours. Absorbance was measured at a reference wavelength of 450 nm. [file 13567_2026_1768_MOESM3_ESM.docx]

**Additional file 3.** Effects of S1PR1-specific agonist SEW2871 and antagonist W146 on DF-1 cell activity. DF-1 cells were seeded at a density of 5 × 10³ cells per well in a 96-well plate. After treatment with W146, SEW2871, or dimethyl sulfoxide (DMSO), 10 μl of CCK-8 reagent was added and incubated at 37°C for 2 hours. Absorbance was measured at a reference wavelength of 450 nm.
