# Supplementary material for: Avian coronaviruses induce inflammatory responses by activating p38/MAPK signaling and NLRP3/caspase-1 inflammasomes through sphingosine-1-phosphate receptor 1
Source: Vet Res. 2026 May 23;57:83. doi: 10.1186/s13567-026-01768-0 (PMC13198749; doi:10.1186/s13567-026-01768-0)
Supplement: Supplementary file 4 — Additional file 4: Enrichment results for Amino acid metabolism pathways. [file 13567_2026_1768_MOESM4_ESM.docx]

**Additional file 4.** Enrichment results for Amino acid metabolism pathways.

| Pathway | control-vs-IBV(94) | Reference(30651) | Rich Factor | P value | Q value | Pathway ID | Level 1 | Level 2 | Metabolite | Compounds | |
| --- | --- | --- | --- | --- | --- | --- | --- | --- | --- | --- | --- |
| Cysteine and methionine metabolism | 2 | 175 | 0.011429 | 0.100882 | 2.50E-01 | ko00270 | Metabolism | Amino acid metabolism | M398T768; M220T235 | C00019+C01118 | |
| Arginine biosynthesis | 1 | 56 | 0.017857 | 0.158154 | 2.98E-01 | ko00220 | Metabolism | Amino acid metabolism | M145T86 | C00064 |  |
| Valine, leucine and isoleucine biosynthesis | 1 | 85 | 0.011765 | 0.230055 | 3.76E-01 | ko00290 | Metabolism | Amino acid metabolism | M120T88 | C00188 |  |
| Alanine, aspartate and glutamate metabolism | 1 | 93 | 0.010753 | 0.248797 | 3.76E-01 | ko00250 | Metabolism | Amino acid metabolism | M145T86 | C00064 |  |
| Lysine biosynthesis | 1 | 111 | 0.009009 | 0.289332 | 3.92E-01 | ko00300 | Metabolism | Amino acid metabolism | M170T91 | C20258 |  |
| Lysine degradation | 1 | 130 | 0.007692 | 0.329773 | 4.07E-01 | ko00310 | Metabolism | Amino acid metabolism | M115T266 | C00489 |  |
| Glycine, serine and threonine metabolism | 1 | 153 | 0.006536 | 0.375691 | 4.31E-01 | ko00260 | Metabolism | Amino acid metabolism | M120T88 | C00188 |  |
| Tryptophan metabolism | 1 | 175 | 0.005714 | 0.416695 | 4.63E-01 | ko00380 | Metabolism | Amino acid metabolism | M206T98 | C05660 |  |
| Tyrosine metabolism | 1 | 208 | 0.004808 | 0.473257 | 5.00E-01 | ko00350 | Metabolism | Amino acid metabolism | M201T85 | C01061 |  |
| Arginine and proline metabolism | 1 | 209 | 0.004785 | 0.474883 | 5.00E-01 | ko00330 | Metabolism | Amino acid metabolism | M398T768 | C00019 |  |

Note: Pathway: Name of the enriched KEGG pathway; Second column: Number of differentially expressed metabolites annotated in KEGG; Reference: Total number of metabolites annotated in the background KEGG pathway; Rich Factor: Ratio of enriched differentially expressed metabolites to background metabolites in the pathway; P value: P-value from enrichment analysis; Pathway ID: KEGG Pathway ID; Metabolite: Metabolite ID; Compounds: Corresponding IDs in the KEGG database.
