# Supplementary material for: Avian coronaviruses induce inflammatory responses by activating p38/MAPK signaling and NLRP3/caspase-1 inflammasomes through sphingosine-1-phosphate receptor 1
Source: Vet Res. 2026 May 23;57:83. doi: 10.1186/s13567-026-01768-0 (PMC13198749; doi:10.1186/s13567-026-01768-0)
Supplement: Supplementary file 5 — Additional file 5: Enrichment results for carbohydrate metabolism pathways. [file 13567_2026_1768_MOESM5_ESM.docx]

**Additional file 5.** Enrichment results for carbohydrate metabolism pathways.

| Pathway | control-vs-IBV(94) | Reference(30651) | RichFactor | P value | Q value | Pathway ID | Level 1 | Level 2 | Metabolite | Compounds |
| --- | --- | --- | --- | --- | --- | --- | --- | --- | --- | --- |
| Glyoxylate and dicarboxylate metabolism | 2 | 181 | 0.01105 | 0.106703 | 2.50E-01 | ko00630 | Metabolism | Carbohydrate metabolism | M145T86;M148T85 | C00064+C00975 |
| Fructose and mannose metabolism | 1 | 119 | 0.008403 | 0.306646 | 3.94E-01 | ko00051 | Metabolism | Carbohydrate metabolism | M259T90 | C00636 |
| Pentose and glucuronate interconversions | 1 | 146 | 0.006849 | 0.362056 | 4.21E-01 | ko00040 | Metabolism | Carbohydrate metabolism | M370T99 | C02273 |
| Amino sugar and nucleotide sugar metabolism | 1 | 267 | 0.003745 | 0.561184 | 5.72E-01 | ko00520 | Metabolism | Carbohydrate metabolism | M259T90 | C00636 |

Note: Pathway: Name of the enriched KEGG pathway; Second column: Number of differentially expressed metabolites annotated in KEGG; Reference: Total number of metabolites annotated in the background KEGG pathway; Rich Factor: Ratio of enriched differentially expressed metabolites to background metabolites in the pathway; P value: P-value from enrichment analysis; Pathway ID: KEGG Pathway ID; Metabolite: Metabolite ID; Compounds: Corresponding IDs in the KEGG database.
