# Supplementary material for: Avian coronaviruses induce inflammatory responses by activating p38/MAPK signaling and NLRP3/caspase-1 inflammasomes through sphingosine-1-phosphate receptor 1
Source: Vet Res. 2026 May 23;57:83. doi: 10.1186/s13567-026-01768-0 (PMC13198749; doi:10.1186/s13567-026-01768-0)
Supplement: Supplementary file 6 — Additional file 6: Enrichment results for lipid metabolism pathways. [file 13567_2026_1768_MOESM6_ESM.docx]

**Additional file 6.** Enrichment results for lipid metabolism pathways.

| Pathway | control-vs-IBV(94) | Reference(30651) | RichFactor | P value | Q value | Pathway ID | Level 1 | Level 2 | Metabolite | Compounds |
| --- | --- | --- | --- | --- | --- | --- | --- | --- | --- | --- |
| Primary bile acid biosynthesis | 3 | 103 | 0.029126 | 0.003958 | 4.40E-02 | ko00120 | Metabolism | Lipid metabolism | M375T809;M515T625;M516T623 | C02528+C05122 |
| Secondary bile acid biosynthesis | 3 | 119 | 0.02521 | 0.005917 | 5.92E-02 | ko00121 | Metabolism | Lipid metabolism | M375T809;M515T625;M516T623 | C02528+C05122 |
| Sphingolipid metabolism | 2 | 71 | 0.028169 | 0.020161 | 1.26E-01 | ko00600 | Metabolism | Lipid metabolism | M378T777;M300T746 | C06124+C12144 |
| Fatty acid degradation | 2 | 103 | 0.019417 | 0.040021 | 2.00E-01 | ko00071 | Metabolism | Lipid metabolism | M115T266;M874T419 | C00489+C05276 |
| Cutin, suberine and wax biosynthesis | 1 | 44 | 0.022727 | 0.126495 | 2.64E-01 | ko00073 | Metabolism | Lipid metabolism | M271T840 | C18218 |
| Biosynthesis of unsaturated fatty acids | 2 | 210 | 0.009524 | 0.135994 | 2.72E-01 | ko01040 | Metabolism | Lipid metabolism | M284T938;M289T632 | C01530+C03242 |
| Fatty acid elongation | 1 | 75 | 0.013333 | 0.205976 | 3.47E-01 | ko00062 | Metabolism | Lipid metabolism | M874T419 | C05276 |
| Linoleic acid metabolism | 1 | 96 | 0.010417 | 0.255708 | 3.76E-01 | ko00591 | Metabolism | Lipid metabolism | M289T632 | C03242 |
| alpha-Linolenic acid metabolism | 1 | 113 | 0.00885 | 0.2937 | 3.92E-01 | ko00592 | Metabolism | Lipid metabolism | M988T458 | C16335 |
| Glycerophospholipid metabolism | 1 | 137 | 0.007299 | 0.344091 | 4.14E-01 | ko00564 | Metabolism | Lipid metabolism | M489T100 | C00307 |
| Fatty acid biosynthesis | 1 | 142 | 0.007042 | 0.354132 | 4.17E-01 | ko00061 | Metabolism | Lipid metabolism | M284T938 | C01530 |
| Steroid hormone biosynthesis | 1 | 207 | 0.004831 | 0.471625 | 5.00E-01 | ko00140 | Metabolism | Lipid metabolism | M332T528 | C18040 |

Note: Pathway: Name of the enriched KEGG pathway; Second column: Number of differentially expressed metabolites annotated in KEGG; Reference: Total number of metabolites annotated in the background KEGG pathway; Rich Factor: Ratio of enriched differentially expressed metabolites to background metabolites in the pathway; P value: P-value from enrichment analysis; Pathway ID: KEGG Pathway ID; Metabolite: Metabolite ID; Compounds: Corresponding IDs in the KEGG database.
